# Brewer’s Spent Grain as a Substrate for Lactic Acid Fermentation: Contrasting Performance of *Lacticaseibacillus rhamnosus* and *Limosilactobacillus fermentum*

**DOI:** 10.3390/foods15203715

**Published:** 2026-10-19

**Authors:** Minh Quoc Ha, Phuc Hanh Nguyen, Allan Billardey, Solene Moulin, Hui Shing Him, Rothman Kam, Brent Seale, Claus Heiner Bang-Berthelsen, Thao T. Le

**Affiliations:** 1 Department of Biomedical, Chemical, and Food Sciences, School of Science, Auckland University of Technology, Auckland 1010, New Zealand; 2 National Food Institute, Technical University of Denmark, DK-2800 Kongens Lyngby, Denmark

**Keywords:** brewer’s spent grain, BSG, solid-state fermentation, *Lacticaseibacillus rhamnosus*, *Limosilactobacillus fermentum*, in vitro bioactivity

## Abstract

Brewer’s spent grain (BSG), the major by-product of the brewing industry, has attracted growing interest in valorisation through lactic acid bacteria fermentation. However, research has largely focused on *Lactiplantibacillus plantarum*, while other species have mainly been investigated using enzymatic hydrolysates rather than intact solid BSG. This study compared the solid-state fermentation performance of *Lacticaseibacillus rhamnosus* ATCC 7469 *and Limosilactobacillus fermentum* ATCC 9338 on BSG over 10 days. *In vitro* antioxidant, antidiabetic, anti-obesity, and antimicrobial activities were assessed every two days and integrated into a global bioactivity score. *Lacticaseibacillus rhamnosus* successfully colonised the substrate, reaching 9.62 log_10_ CFU g^−1^ on Day 2 and reducing the pH to 3.6. Antioxidant capacity peaked on Day 2, whereas antidiabetic and anti-obesity activities peaked on Day 10. Bactericidal activity was also confirmed in a bread-simulating matrix, and the global bioactivity score identified Day 2 as the highest-scoring endpoint. In contrast, *Limosilactobacillus fermentum* failed to establish a successful fermentation, with polymicrobial contamination observed from Day 4 onwards. To investigate whether substrate sterilisation could improve *Limosilactobacillus fermentum* fermentation performance, autoclaving and a two-step heat treatment were evaluated. Although autoclaving effectively sterilised the substrate, it reduced the number of detectable proteins by nearly 25%, whereas the two-step heat treatment preserved the protein profile but did not support successful fermentation by *Limosilactobacillus fermentum*. Overall, these findings identify *Lacticaseibacillus rhamnosus* as a promising candidate for BSG valorisation and underscore the importance of strain selection, as fermentation performance can vary substantially among lactic acid bacteria.

## 1. Introduction

Beer is one of the most widely consumed beverages worldwide, and its production generates large volumes of brewer’s spent grain (BSG), the most abundant brewing by-product, with an estimated annual production of 39 million tonnes [1]. BSG is highly perishable and is used predominantly as animal feed or otherwise disposed of as a low-value by-product, creating both economic and environmental incentives for higher-value utilisation [1,2]. Consequently, increasing attention has been directed towards valorising BSG as a functional food ingredient rather than treating it as a waste stream.

The potential of BSG as a functional ingredient arises from its nutritional and bioactive composition. It contains dietary fibre, storage proteins such as hordeins and glutelins, and bioactive polyphenols present in relatively low concentrations [3,4,5]. Nevertheless, many of these bioactive compounds are entrapped within the grain’s lignocellulosic matrix, where phenolic compounds and protein-bound bioactive precursors exhibit limited solubility and bioavailability [6]. Thus, targeted processing strategies capable of disrupting this matrix are required to unlock the full functional potential of BSG.

Two main strategies have been investigated to disrupt this matrix and release the bound compounds: enzymatic hydrolysis and fermentation. Enzymatic hydrolysis can markedly increase bioactivity; for example, protease treatment has been reported to raise the antioxidant capacity of BSG by more than threefold [7]. However, this approach has limitations including the high cost of enzymes and limited peptide diversity arising from enzyme substrate specificity [8]. Fermentation with lactic acid bacteria (LAB) provides an alternative approach, as the microorganisms produce their own enzymes during fermentation, eliminating the need for commercial preparations. Their combined proteolytic and metabolic activities generate a broader range of bioactive compounds with enhanced antioxidant, antidiabetic, and antimicrobial properties [6,9]. Among LAB species, BSG fermentation research has largely centred on *Lactiplantibacillus plantarum* due to its natural association with plant material and its unusually large and flexible genome. It carries numerous gene clusters for carbohydrate utilisation, which allow growth on the diverse sugars present in plant substrates [10,11]. Our recent studies have investigated BSG fermentation using this species, characterising peptide release over a 10-day fermentation period [5] and comparing aerobic and anaerobic processing conditions [12]. However, both studies were limited to a single LAB species known to successfully ferment BSG and thus did not address the ability of other LAB species to colonise and utilise this substrate.

The two alternative strains examined in this study were *Lacticaseibacillus rhamnosus* (*L. rhamnosus*) and *Limosilactobacillus fermentum* (*L. fermentum*). The former is a well-characterised probiotic that produces proteolytic enzymes and bioactive metabolites during fermentation of dairy and cereal substrates [13,14], whereas the latter shows proteolytic and amylolytic activities in cereal-based substrates [15]. These properties are relevant because the principal fermentable components of BSG are residual protein and starch, and the ability to hydrolyse these fractions would be expected to support microbial growth. Given the marked differences in the enzymatic and metabolic capacities of LAB species, the bioactivities generated during fermentation may vary substantially between strains [16]. Both species have previously been investigated in BSG fermentations; however, the BSG was first enzymatically hydrolysed and subsequently fermented under submerged conditions, with lactic acid production rather than bioactivity generation as the primary objective [13,17]. Hence, their ability to ferment intact BSG and generate bioactive properties under solid-state fermentation remains poorly characterised.

To address these gaps, the present study evaluated and compared the fermentation performance of *L. rhamnosus* and *L. fermentum* on BSG during a 10-day solid-state fermentation, with sampling every two days. Fermented BSG extracts were assessed using four *in vitro* assays: (1) antioxidant activity, measured by ferric reducing antioxidant power (FRAP) and 2,2-diphenyl-1-picrylhydrazyl (DPPH) radical scavenging assays; (2) antidiabetic activity, measured by α-glucosidase inhibition; (3) anti-obesity activity, measured by lipase inhibition; and (4) antimicrobial activity. These endpoints were selected because they represent the bioactivities most frequently reported for LAB-fermented BSG [9], and encompass both health-promoting effects associated with metabolic disorders and preservative functions relevant to food applications. A global bioactivity score was used to integrate all assay results into a single index and identify the fermentation endpoint with the greatest overall bioactivity. The corresponding extract was then evaluated for antimicrobial activity in milk- and wheat-based food model systems. In addition, when a strain failed to establish a successful fermentation, heat pretreatment of the substrate was evaluated as a strategy to improve fermentation performance. Filter-aided sample preparation (FASP)-based proteomics was subsequently used to characterise treatment-induced changes in protein composition and to determine whether these changes could help explain the observed fermentation outcomes.

## 2. Materials and Methods

### 2.1. Chemicals

All chemical reagents were commercially sourced. Tris-HCl, Trolox, 2,4,6-tris(2-pyridyl)-s-triazine (TPTZ), 2,2-diphenyl-1-picrylhydrazyl (DPPH), sodium acetate, glacial acetic acid, hydrochloric acid (37%), iron (III) chloride hexahydrate, sodium carbonate (Na_2_CO_3_), and enzymes including protease from *Aspergillus oryzae* (Cat. No. P6110, density 1.10 to 1.30 g/mL), α-amylase from *Aspergillus oryzae* (Cat. No. A9857), and α-glucosidase from *Saccharomyces cerevisiae* (Cat. No. G5003) were obtained from Sigma-Aldrich (St. Louis, MO, USA). 4-Nitrophenyl acetate (p-NPA, Cat. No. J98614) was acquired from AK Scientific (Union City, CA, USA). Dimethyl sulfoxide (DMSO, ≥99%, reagent grade) and acetonitrile (LC-MS grade) were sourced from Thermo Fisher Scientific (Waltham, MA, USA), while Lipase AY30 (Cat. No. AC294760250) was obtained from Acros Organics, Thermo Fisher Scientific (Morris Plains, NJ, USA).

### 2.2. Bacterial Strains

For BSG fermentation, two LAB strains were obtained from the American Type Culture Collection (ATCC, Manassas, VA, USA): *L. rhamnosus* ATCC 7469 and *L. fermentum* ATCC 9338. Both strains were activated according to the supplier’s instructions. Briefly, freeze-dried cultures were resuspended in De Man, Rogosa and Sharpe (MRS) broth and incubated aerobically for 48 h prior to use.

For antimicrobial assays, *Escherichia coli* (*E. coli*, NZRM 916), *Staphylococcus aureus* (*S. aureus*, NZRM 917), and *Bacillus cereus* (*B. cereus*, NCTC 8035) were used as indicator bacteria. Culture conditions for these bacteria will be described in Section 2.5.1.

Culture media and cultivation supplies, including Mueller Hinton (MH) medium, MRS broth and agar, and BD GasPak™ EZ Anaerobe Container System sachets, were purchased from Fort Richard Laboratories Ltd. (Auckland, New Zealand).

### 2.3. Sample Preparation, Fermentation and Extraction Methods

#### 2.3.1. Preparation of BSG

Wheat BSG was obtained from beer brewed for teaching purposes in the Food Laboratory, School of Science, Auckland University of Technology, New Zealand, following the procedure described in our previous study [5]. Each batch was brewed using approximately 2.5 kg of grain, comprising Pilsner malt (75%; Simpsons Malt, Northumberland, United Kingdom), white wheat malt (7.5%; Brewshop, Hamilton, New Zealand), Munich malt (8.5%; Weyermann Specialty Malts, Bamberg, Germany), and dextrin malt (8.5%; Simpsons Malt, Northumberland, UK). Briefly, the grains were mashed at 67 °C for 60 min, followed by sparging with hot water at 75 °C. The resulting wort (approximately 10 L) was used for beer fermentation, and the residual BSG was collected, freeze-dried, ground into a powder, and stored in vacuum-sealed bags at −20 °C until further use.

#### 2.3.2. Fermentation Procedure

Both strains were cultivated at 35 °C under aerobic conditions for 48 h. The same temperature was maintained during subsequent BSG solid-state fermentation, avoiding a temperature shift between culture preparation and fermentation and ensuring consistency with our previous BSG fermentation work [5]. In addition, 35 °C falls within the typical growth range reported for many mesophilic lactic acid bacteria [18]. These conditions were selected to facilitate a direct comparison of strain performance under identical conditions rather than to optimise the growth of each strain individually. After incubation, the cultures were centrifuged at 13,000 rpm for 2 min and washed twice with 0.9% saline. The resulting pellets were resuspended in Milli-Q water and adjusted to an optical density at 600 nm (OD_600_) of 0.6, corresponding to a 3 McFarland standard (approximately 9 × 10^8^ colony-forming units (CFU)/mL)). The bacterial suspensions were then mixed with BSG at a ratio of 3:2 (*w*/*v*), corresponding to 3 g of BSG per 2 mL of inoculum suspension. Fermentation was carried out under aerobic conditions at 35 °C for 10 days, with samples collected every 2 days for cell enumeration on MRS agar and pH measurement. Day 0 samples were collected immediately after inoculation and before incubation and therefore represented an inoculated baseline rather than uninoculated BSG. They were subjected to the same extraction and analytical procedures as all subsequent time points and served as the reference for statistical comparison. In parallel, an uninoculated control was prepared in the same manner, with sterile water replacing the bacterial suspension at the same 3:2 (*w*/*v*) ratio. The control was incubated alongside the inoculated fermentations under identical conditions and sampled at the same six time points. It underwent the same extraction and analytical procedures and served as the time-matched uninoculated reference for both strains. The two references served different purposes. Day 0 defines the starting point of each fermentation and provided the baseline for temporal changes, whereas the uninoculated control captured changes in the substrate during incubation in the absence of deliberate inoculation.

#### 2.3.3. Extraction Method

BSG extracts were prepared by mixing 0.1 g of fermented BSG, on a wet-weight basis, with 1.5 mL of 60% (*v*/*v*) acetonitrile in water. The mixture was sonicated for 30 min in a sonicator bath (UltrasonicsNZ, ULDSL-4, Napier, New Zealand), followed by centrifugation at 10,000 × *g* for 30 min at 10 °C. A 1 mL aliquot of the supernatant, corresponding to 0.06667 g of BSG, was transferred to a fresh tube and dried to completion using a vacuum concentrator (Concentrator Plus, Eppendorf AG, Hamburg, Germany). For antioxidant assays, the acetonitrile extracts were used directly. For the remaining bioactivity assays, the dried extract was reconstituted in an appropriate assay medium. Specifically, the extract was reconstituted in 200 µL of MH broth or food-model medium for the antimicrobial assays, providing a starting concentration of 333.3 mg BSG equivalent/mL, and in 100 µL of the corresponding buffer for the antidiabetic and anti-obesity assays, providing 666.7 mg BSG equivalent/mL. Concentrations are expressed as BSG equivalents, defined as the mass of fermented BSG represented by the extract present in 1 mL of assay medium, rather than the mass of the dried extract itself.

### 2.4. Antioxidant Assay

The antioxidant activity of fermented BSG extracts was measured using FRAP and DPPH assays.

#### 2.4.1. FRAP Assay

The FRAP assay was performed according to the method described by Permal et al. [19]. Trolox standards (5–160 mg/L in 75% ethanol) were used to generate a calibration curve (R^2^ = 0.9996). The FRAP reagent was freshly prepared by mixing TPTZ (0.01 M), FeCl_3_·6H_2_O (0.02 M), and acetate buffer (0.3 M) at a ratio of 1:1:10. For the assay, 0.1 mL of extract or Trolox standard was mixed with 2 mL of FRAP reagent and 0.9 mL of distilled water. After incubation at room temperature for 5 min, absorbance was measured at 593 nm using Tecan Infinite M1000 Pro (Tecan Group Ltd., Männedorf, Switzerland). A blank consisting of 2 mL of FRAP reagent and 1 mL of distilled water was included. Antioxidant activity was expressed as mg Trolox equivalent antioxidant capacity (TEAC) per g of BSG.

#### 2.4.2. DPPH Assay

The DPPH assay followed the method described by Nongonierma and FitzGerald [20], with some modifications. Trolox standards were prepared in 75% ethanol at concentrations ranging from 12.5 to 100 mg/L, yielding a calibration curve with an R^2^ of 0.9996. Briefly, 50 µL of extract or Trolox standard was added to a 96-well plate and mixed with 150 µL of methanolic DPPH solution (0.2 mM). The control consisted of 50 µL of 75% ethanol and 150 µL of DPPH solution, while a blank contained 200 µL of 75% ethanol. The plate was covered with foil and incubated at room temperature for 20 min. Absorbance was subsequently measured at 517 nm using a Tecan Infinite M1000 Pro microplate reader. DPPH radical scavenging activity was calculated using Equation (1) and expressed as mg TEAC per g of BSG.

(1) $$\mathrm{Inhibition}\;\mathrm{of}\;\mathrm{DPPH}(\%)=(\frac{\mathrm{Absorbance}\;\mathrm{of}\;\mathrm{control}-\mathrm{Absorbance}\;\mathrm{of}\;sample}{\mathrm{Absorbance}\;\mathrm{of}\;\mathrm{control}})\;\times \;100$$

### 2.5. Antimicrobial Assay

#### 2.5.1. *In Vitro* Antimicrobial Activity

The antimicrobial activity of fermented BSG extracts was assessed by broth microdilution to determine the minimum inhibitory concentration (MIC) and minimum bactericidal concentration (MBC) values. *S. aureus* (NZRM 917), *B. cereus* (NCTC 8035), and *E. coli* (NZRM 916) were tested following Aurori et al. [21]. Bacteria were cultured in MH broth at 35 °C for 24 h, then diluted to approximately 10^5^ CFU/mL. Initial colony counts were performed to confirm inoculum concentration.

Dried extracts were reconstituted in 200 µL of MH broth to give a stock concentration of 333.3 mg BSG/mL. From this stock, 100 µL was added to the first well of a 96-well plate and subjected to two-fold serial dilution in MH broth, yielding concentrations of 333.3, 166.7, 83.3, and 41.7 mg BSG/mL at a final volume of 100 µL per well. Each well was inoculated with 5 µL of bacterial suspension, giving a final volume of 105 µL and approximately 500 CFU per well, and plates were incubated at 35 °C for 24 h. A growth control consisting of inoculated MH broth without extract was included on every plate. MIC was defined as the lowest concentration with no visible turbidity compared with the inoculated control [21].

For MBC determination, 10 µL from the MIC well and the two higher concentrations were plated on MH agar and incubated for 24 h. The MBC was defined as the lowest concentration at which no colonies were detected after subculture, corresponding to a detection limit of fewer than 10 viable cells per well and a reduction of at least 98% relative to the initial inoculum. The actual reduction may have been greater, as the assay could not resolve counts below this limit. The MIC index, calculated as MBC/MIC, was used to classify activity as bactericidal when ≤4 or bacteriostatic when >4 [22].

#### 2.5.2. Antimicrobial Activity in Food-Model Media

Antimicrobial activity in food-model media was tested in two food-simulating media: bread-based medium, representing wheat-based products, and skim milk medium, representing dairy products.

Bread-based medium: The bread-based medium was prepared following Iosca et al. [23], with minor modifications. Commercial wheat bread (Woolworths, Auckland, New Zealand) was homogenised with water at a 1:4 ratio (*w*/*w*). Protease and α-amylase were added at 1 mL/L and 0.5 g/L, respectively. The mixture was incubated at 55 °C for 18 h, filtered through Whatman filter paper, adjusted to pH 7, and sterilised at 121 °C for 15 min. After sterilisation, it was centrifuged at 3200× *g* for 20 min to remove sediment.

Skim milk medium: The skim milk medium was prepared according to Tayel et al. [24]. Skim milk powder (Woolworths, Auckland, New Zealand) was dissolved in distilled water at a 1:10 ratio (*w*/*v*), following the manufacturer’s instructions. The medium was autoclaved at 121 °C for 15 min before use.

Antimicrobial activity in food-model media assay: Extracts exhibiting bactericidal activity in the *in vitro* assay were selected for food-model media testing. Assays were performed in 96-well plates using sterile food-based media for dilution. Each extract was subjected to two-fold serial dilution to obtain final concentrations of 333.3, 166.7, 83.3, and 41.7 mg BSG/mL, inoculated with 5 µL of bacterial suspension (10^5^ CFU/mL), and incubated at 35 °C for 24 h. MBC was determined as described in Section 2.5.1. All assays were performed in triplicate.

### 2.6. Antidiabetic (α-Glucosidase Inhibition) Assay

α-Glucosidase inhibition was measured following Telagari & Hullatti [25], with minor modifications. Each well contained 50 µL of phosphate buffer (100 mM, pH 6.8), 10 µL of α-glucosidase (1 U/mL), and 20 µL of extract. After pre-incubation at 37 °C for 10 min, 20 µL p-nitrophenyl-α-D-glucopyranoside (p-NPG, 5 mM) was added. The plate was incubated for another 20 min at 37 °C, and the reaction was stopped by adding 50 µL of Na_2_CO_3_ (0.1 M). The control contained 20 µL of buffer instead of the extract. Absorbance was measured at 405 nm using a Tecan Infinite M1000 Pro microplate reader. Results were expressed as mg acarbose equivalents (AE) per g BSG, based on the IC_50_ of acarbose (0.93 mg/mL) [25]. Inhibition percentage was calculated using Equation (2):


(2) $$\mathrm{Inhibition}\;(\%)=(\frac{\mathrm{Absorbance}\;\mathrm{of}\;\mathrm{control}-\mathrm{Absorbance}\;\mathrm{of}\;sample}{\mathrm{Absorbance}\;\mathrm{of}\;\mathrm{control}})\;\times \;100$$

### 2.7. Anti-Obesity (Anti-Lipase) Assay

Lipase inhibition was measured following Zhang et al. [26]. Lipase was prepared by dissolving 3 mg of enzyme in 1 mL of Tris-HCl buffer (13 mM, pH 8.5) containing CaCl_2_ (1.3 mM) and NaCl (150 mM). The solution was vortexed for 5 min and centrifuged at 3200 × *g* for 15 min at 25 °C, and the supernatant was collected and used as the enzyme solution.

In a 96-well plate, 50 µL of extract was mixed with 50 µL enzyme solution and incubated at 37 °C for 10 min. Subsequently, 50 µL of p-NPA solution (2 mg/mL in DMSO) was added, followed by incubation at 37 °C for 20 min. The control contained 50 µL of buffer in place of the extract. Absorbance was then measured at 405 nm. Results were expressed as mg orlistat equivalents (OE) per g BSG, based on the IC_50_ of orlistat (0.55 mg/mL) [26]. Inhibition percentage was calculated using Equation (3):


(3) $$\mathrm{Inhibition}\;(\%)=(\frac{\mathrm{Absorbance}\;\mathrm{of}\;\mathrm{control}-\mathrm{Absorbance}\;\mathrm{of}\;sample}{\mathrm{Absorbance}\;\mathrm{of}\;\mathrm{control}})\;\times \;100$$

### 2.8. Investigation of Heat Pretreatment on BSG

#### 2.8.1. BSG Heat Pretreatment

To improve the colonisation of *L. fermentum* on BSG, substrate sterilisation was investigated as a potential strategy to reduce microbial competition from the native microbiota. Two heat treatment methods (two-step heat treatment and autoclave treatment) were therefore applied to BSG before inoculation.

For two-step heat treatment, powdered BSG was treated using a protocol adapted from Løvdal et al. [27]. Samples were first heated in a water bath to an internal temperature of 70 °C, confirmed with a calibrated thermometer, and held for 17 min. They were then incubated at 30 °C for 60 min to allow surviving spores to germinate, followed by a second heat treatment at 80 °C for 12 min to inactivate all the germinated cells. For autoclave treatment, BSG was sterilised at 121 °C for 15 min in a standard laboratory autoclave.

Following pretreatment, four fermentation groups were established to evaluate the fermentation performance of *L. fermentum*: autoclaved BSG inoculated with *L. fermentum* (AF), two-step heat-treated BSG inoculated with *L. fermentum* (TF), autoclaved BSG inoculated with *L. rhamnosus* (AR), and uninoculated two-step heat-treated BSG (CT). The AR group served as a positive control to verify that the sterilised substrate remained capable of supporting LAB growth, and the CT group as a sterility control to test whether the milder treatment eliminated the native microbiota. Inoculation was performed using the same bacterial preparation and substrate-to-inoculum ratio described in Section 2.3.2. Fermentation was conducted at 35 °C and samples collected on Days 0, 4, and 10 for pH measurement and microbial enumeration on MRS agar.

#### 2.8.2. Bioactivity of Heat-Treated and Fermented BSG

Bioactivity was assessed only in the AF and TF groups, as these treatments were designed to determine whether heat pretreatment could restore the fermentation capacity of *L. fermentum*. Samples collected on Days 0, 4, and 10 were extracted as described in Section 2.3.3, with the Day 0 samples representing heat-treated BSG collected immediately after inoculation and before incubation. Antioxidant activity was determined by FRAP and DPPH assays (Section 2.4), antidiabetic activity by α-glucosidase inhibition (Section 2.6), and anti-obesity activity by lipase inhibition (Section 2.7).

#### 2.8.3. Proteomics Analysis of BSG

##### Tryptic Digestion

To characterise the effect of heat treatment on BSG protein composition, FASP-based proteomics was performed on uninoculated (raw BSG), two-step heat-treated, and autoclaved BSG following the protocol of Ha et al. [5], with minor modifications. A 45 mg sample of BSG slurry containing 4 mg protein was used for tryptic digestion. Briefly, BSG samples were extracted in 1 mL of lysis buffer (8 M urea, 0.1 M Tris-HCl, 2% DTT) at 600 rpm for 30 min at 20 °C and centrifuged (10,000× *g*, 15 min, 10 °C). Protein extracts (100 µL) were loaded onto 10 kDa MWCO filters (Merck Millipore, Bayswater, VIC, Australia), washed twice with urea buffer (8 M urea in 0.1 M Tris-HCl), and alkylated with 25 mM iodoacetamide for 20 min at 20 °C in the dark. Filters were subsequently washed twice with urea buffer and twice with 50 mM ammonium bicarbonate. Proteins were digested with trypsin (0.02 µg/µL in 50 mM ammonium bicarbonate) at 37 °C with shaking at 800 rpm for 16 h. Digestion was terminated with 1% formic acid, and peptides were recovered by centrifugation (20,000× *g*, 30 min, 10 °C), vacuum-dried, stored at −80 °C, and reconstituted in 0.1% formic acid prior to analysis.

##### LC-MS/MS

Tryptic peptides were analysed by nano-liquid chromatography coupled to tandem mass spectrometry (nanoLC-MS/MS) using a Vanquish Neo nanoUHPLC system coupled to an Orbitrap Exploris 240 mass spectrometer (Thermo Fisher Scientific, Bremen, Germany). The dried digests were reconstituted in 100 µL of 0.1% formic acid and injected at 1 µL per run using a trap-and-elute workflow in NanoCap mode, with the autosampler maintained at 7 °C. Chromatographic separation was performed on a Thermo EASY-Spray PepMap Neo C18 reversed-phase analytical column (75 µm × 150 mm, 2 µm) at an analytical flow rate of 0.250 µL/min. Mobile phase A consisted of water containing 0.1% formic acid and mobile phase B consisted of 80% acetonitrile containing 0.1% formic acid. Peptides were separated using a 60 min gradient: 4% B held from 0 to 1 min, ramping linearly to 25% B at 42 min, then to 40% B at 51 min, followed by a column wash at 100% B from 53 to 60 min at an increased flow rate of 0.500 µL/min.

The mass spectrometer was operated in positive ion mode using data-dependent acquisition (DDA). Full scan MS1 spectra were acquired in the Orbitrap over *m/z* 375–1200 at a resolution of 60,000 (FWHM at *m/z* 200), with an AGC target of 3.0 × 10^6^ and automatic maximum injection time. Precursor ions with charge states between 2 and 4 and a minimum intensity threshold of 1.0 × 10^4^ counts were selected for fragmentation using higher-energy collisional dissociation at a normalised collision energy of 28%, with a 2 *m/z* isolation window. MS2 spectra were acquired in the Orbitrap at a resolution of 15,000, with a first mass threshold of *m/z* 120, an AGC target of 5.0 × 10^4^, and dynamic exclusion applied for 20 s after a single observation within ±10 ppm mass tolerance. A data-dependent cycle time of 1.5 s was applied between consecutive full scans.

##### Data Processing

Raw MS data were processed using Thermo Proteome Discoverer 3.1 and searched against a combined protein sequence database comprising *Hordeum vulgare*, *Triticum aestivum*, and *Avena sativa* entries, supplemented with reviewed sequences from *L. rhamnosus* and *L. fermentum* (UniProt Knowledgebase, September 2025), common laboratory contaminants from the cRAP repository, and trypsin (UniProt P00760). Cysteine carbamidomethylation was applied as a fixed modification; methionine oxidation and protein N-terminal acetylation were set as variable modifications. Trypsin was specified as the digestion enzyme with a maximum of two missed cleavages. Precursor and fragment ion mass tolerances were set to 10 ppm and 0.02 Da, respectively. Peptide and protein identifications were filtered to a false discovery rate of 1% at both the peptide-spectrum match and protein levels using the Percolator node. Proteins detected in raw BSG but absent after autoclaving were assigned to functional categories based on keyword matching of their UniProt annotations using a custom Python script (Python 3.13.7; pandas 3.0.1; NumPy 2.4.3).

### 2.9. Global Bioactivity Score

To summarise overall functionality, a global bioactivity score was calculated from the individual assay results. Because lower MBC values indicate stronger activity, MBC values were multiplied by −1 so that higher values indicated greater activity in every assay. All assays were then standardised as z-scores across the dataset and averaged within domains: FRAP and DPPH as an antioxidant index, and the three test organisms as an antimicrobial index. The global bioactivity score was calculated as the unweighted mean of the four domain indices, each contributing 25%, and therefore reflects relative differences among the samples in this study rather than absolute bioactivity. Raw values, z-scores, and the resulting scores for every replicate are provided in Table S1. To assess the robustness of the scoring approach, two sensitivity analyses were performed. First, the score was recalculated after removing each of the four bioactivity domains in turn to determine whether any single domain disproportionately influenced the ranking of fermentation days (Table S2A). Second, the assigned MBC value was replaced with 500, 1000, or 1333.3 mg BSG equivalent/mL to evaluate the sensitivity of the score to the imputed value of 666.7 mg BSG equivalent/mL (Table S2B). Scores were rescaled to 0–1 for plotting only, and all statistical analyses were performed using the untransformed values.

### 2.10. Statistical Analysis

Inoculated fermentations were performed as three independent biological replicates and are reported as mean ± standard deviation (SD); the uninoculated control was prepared in duplicate and is presented as a descriptive reference only, with no statistical testing (Figures S1 and S2; Table S3). Day 0 was compared with each subsequent time point using two-sided Welch’s two-sample *t*-tests for both the individual assays and the global bioactivity score. Results for the individual assays are reported using unadjusted *p*-values, with Šídák-adjusted values provided in Table S4; the global bioactivity score is reported using Šídák-adjusted *p*-values across the five comparisons. Statistical significance was accepted at *p* ≤ 0.05. Analyses were performed in Python 3.13.7 (NumPy 2.4.3, pandas 3.0.1 and SciPy 1.17.1), and figures were prepared with Matplotlib 3.10.8.

## 3. Results and Discussion

### 3.1. Fermentation Pattern: pH and Microbial Growth

**Figure 1:**
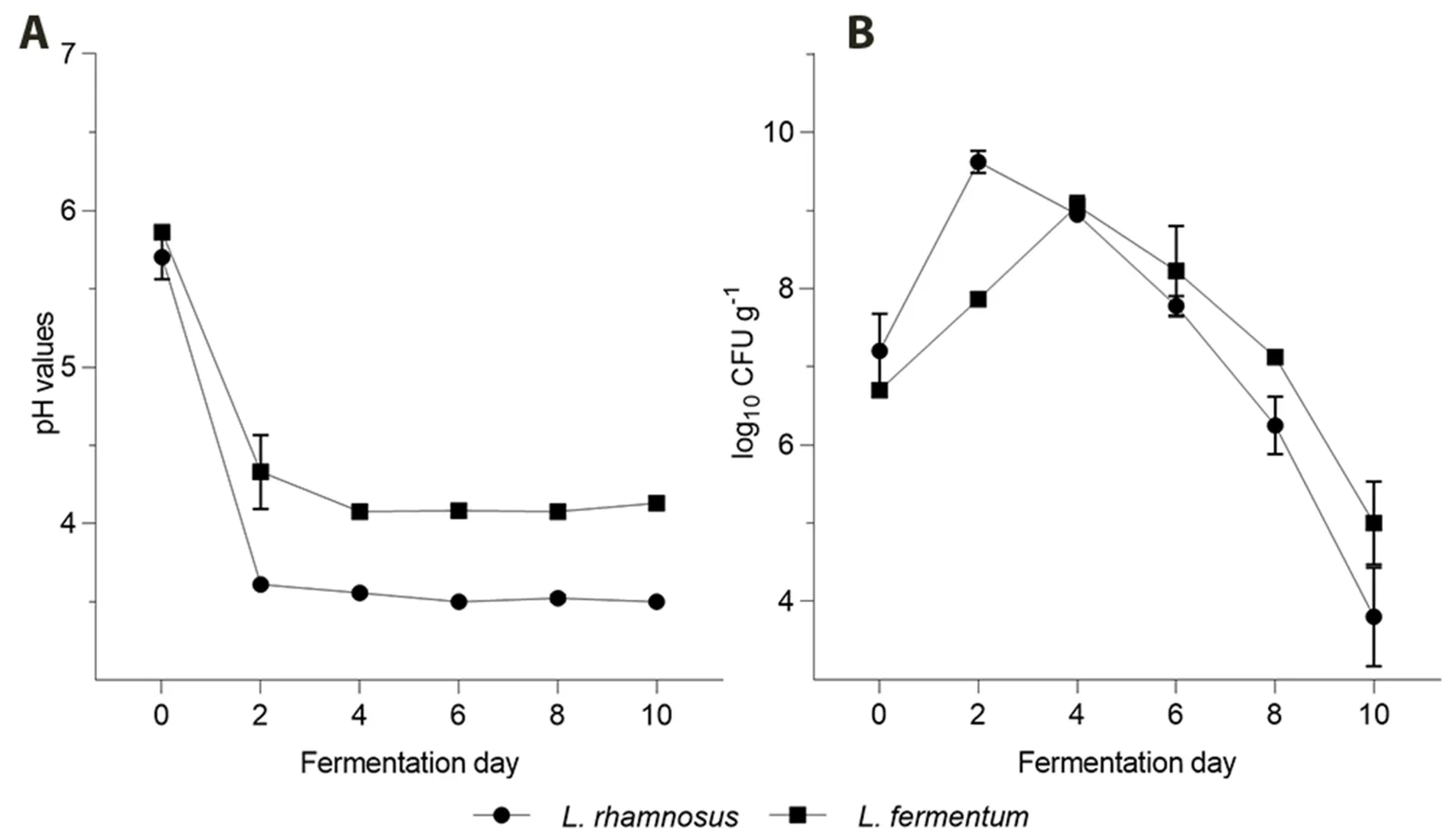
pH changes (**A**) and bacterial growth (**B**) during a 10-day fermentation of brewer’s spent grain (BSG) inoculated with *Lacticaseibacillus rhamnosus* (*L. rhamnosus* ●) and *Limosilactobacillus fermentum* (*L. fermentum* ■). Data are presented as mean ± SD from three independent biological replicates (*n* = 3).

The fermentation of *L. rhamnosus* and *L. fermentum* on BSG over 10 days is illustrated in Figure 1, which presents both pH changes (Figure 1A) and microbial growth profiles of fermented BSG (Figure 1B). Generally, both strains showed changes in pH and viable counts following inoculation; however, their fermentation behaviours differed considerably. *L. rhamnosus* demonstrated a clear acidification and growth pattern, whereas the performance of *L. fermentum* was affected by the emergence of polymicrobial contamination during fermentation.

The two strains began fermentation at similar initial pH values, 5.70 for *L. rhamnosus* and 5.86 for *L. fermentum*. However, their acidification patterns differed clearly from Day 2 onwards. *L. rhamnosus* showed a rapid pH decrease, reaching 3.6 on Day 2 and remaining near 3.5 from Day 4 to Day 10. In contrast, *L. fermentum* displayed weaker acidification, with pH decreasing to 4.3 on Day 2 and then stabilising at around 4.1 for the remainder of the fermentation period (Figure 1A). The uninoculated control also underwent substantial acidification, with pH declining from 5.93 to 3.99 by Day 10 (Figure S1A). Notably, its final pH was lower than that achieved in the *L. fermentum*-inoculated fermentation.

The rapid and sustained acidification by *L. rhamnosus* indicates efficient lactic acid production and effective carbohydrate utilisation within the BSG matrix. The pH decrease observed in this study was greater than that reported previously for the same species fermenting cereal and grain-based substrates, where pH values typically decrease to 4.5 to 4.7 within the first 48 h [13,27]. Several factors may have contributed to this pronounced difference in acidification. First, BSG may have a lower buffering capacity than intact cereal matrices, allowing microbial acid production to cause a greater decrease in pH. Second, the local accumulation of organic acids within the solid substrate may have further contributed to the observed pH decline, as reported for solid-state fermentation systems [11,27]. Together, these factors may explain the rapid and pronounced pH decline observed for *L. rhamnosus*. Although pH measurements alone do not allow the relative contributions of acid production, substrate utilisation, and buffering capacity to be determined, the sustained decrease in pH is consistent with active fermentation.

The different pH trend observed in *L. fermentum* may reflect its metabolic type. *L. fermentum* is obligately heterofermentative, whereas *L. rhamnosus* is facultatively heterofermentative [28]. Obligately heterofermentative LAB metabolise hexoses through the phosphoketolase pathway, producing lactic acid together with carbon dioxide, acetate, and ethanol, whereas *L. rhamnosus* can metabolise hexoses predominantly through glycolysis, resulting in a higher theoretical yield of lactic acid per mole of hexose consumed [28]. Under equivalent hexose consumption, *L. fermentum* would be expected to generate less lactic acid than *L. rhamnosus*. This metabolic difference may partly explain the weaker acidification observed for *L. fermentum* and its higher final pH of 4.1 compared with 3.5 for *L. rhamnosus*.

The microbial growth profiles also differed between the two strains (Figure 1B). *L. rhamnosus* increased from an initial count of 7.2 log_10_ CFU g^−1^ to a maximum of 9.62 log_10_ CFU g^−1^ on Day 2. Cell numbers then declined gradually between Days 4 and 8, followed by a sharp decrease to 3.8 log_10_ CFU g^−1^ on Day 10. *L. fermentum* started at a slightly lower initial count of 6.7 log_10_ CFU g^−1^ and appeared to increase more slowly, reaching 9.0 log_10_ CFU g^−1^ on Day 4. However, from Day 4 onwards, visual and microbiological observations showed morphologically diverse colonies on MRS agar and on the BSG substrate. These included moulds and colony morphotypes that were inconsistent with typical *L. fermentum* morphology (Figure S3), indicating polymicrobial contamination. Consequently, viable cell counts from Day 4 onwards likely represent a mixed microbial population rather than *L. fermentum* growth alone. The uninoculated control carried a native microbiota that was undetectable on Day 0 but reached 8.46 log_10_ CFU g^−1^ by Day 6 (Figure S1B), confirming that untreated BSG supports spontaneous microbial growth under these conditions.

In addition, the decline in viable cell counts of *L. rhamnosus* from Day 4 onwards, following a peak on Day 2,may reflect self-inhibition, as the increasingly acidic environment generated during fermentation can become unfavourable to the organism producing it [18]. In contrast, *L. fermentum* appeared to increase more slowly and reached its maximum two days later, on Day 4. This slower apparent growth may reflect the limited accessibility of the BSG substrate, as arabinoxylans, cellulose, and cell-wall-associated components may not be readily accessible to LAB without prior pretreatment [3,29]. The weaker acidification by *L. fermentum* may also have left the substrate more vulnerable to microbial invasion. Rapid acid production is a major mechanism by which LAB suppress competing microorganisms in fermented substrates [30,31]. Although a direct causal relationship cannot be established, the appearance of polymicrobial contamination from Day 4 onwards coincided with the period during which acidification by *L. fermentum* was least pronounced. Insufficient acidification may thus have reduced the microbial stability of the fermentation and allowed other microorganisms to persist or proliferate. The uninoculated control provides additional evidence that the fermentation was not primarily driven by *L. fermentum.* Despite receiving no starter culture, the uninoculated control reached a viable count comparable to that of the *L. fermentum* treatment and reduced the pH further than the inoculated substrate (Figure 1A and Figure S1). Together, these observations suggest that *L. fermentum* did not establish a fermentation pattern clearly distinguishable from that generated by the native microbiota.

Overall, these results suggest that *L. rhamnosus* was well adapted to BSG fermentation under the conditions tested. In contrast, *L. fermentum* showed lower substrate utilisation, as evidenced by weaker acidification, slower growth kinetics, and the emergence of polymicrobial contamination from Day 4 onwards. These findings highlight the strain- and substrate-specific nature of LAB fermentation, and successful colonisation cannot be assumed based solely on genus-level classification [11,32]. Because the microbiological integrity of *L. fermentum* fermentations was compromised after Day 4, the bioactivity results for the two strains are presented and discussed separately in the following sections. Accordingly, bioactivity data obtained from *L. fermentum* fermentations should be regarded as exploratory and interpreted with caution due to the presence of mixed-community contamination.

### 3.2. Bioactivity Screening of L. rhamnosus Fermented BSG

**Figure 2:**
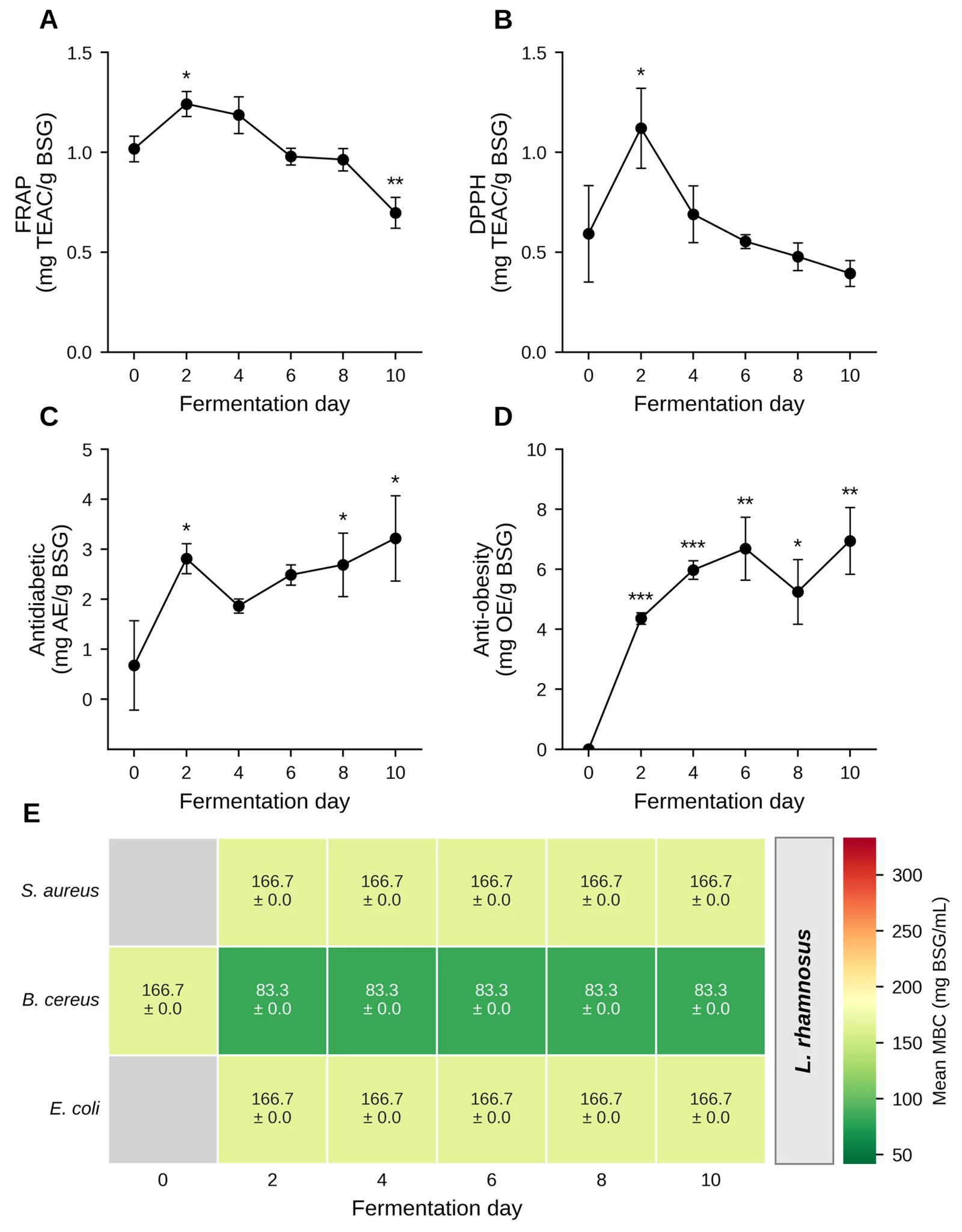
Time-course changes in bioactivity of *L. rhamnosus*-fermented brewer’s spent grain (BSG) extracts over a 10-day period. (**A**,**B**) Antioxidant activity measured by FRAP and DPPH, respectively, expressed as mg TEAC/g BSG. (**C**) Antidiabetic activity, expressed as mg AE/g BSG. (**D**) Anti-obesity activity, expressed as mg OE/g BSG. (**E**) Antimicrobial activity as mean minimum bactericidal concentration (MBC, mg BSG/mL) against *Staphylococcus aureus* (*S. aureus*), *Bacillus cereus* (*B. cereus*), and *Escherichia coli* (*E. coli*) across the fermentation period; lower MBC values indicate stronger antibacterial activity. Grey cells indicate no bactericidal effect within the tested concentration range. Data are presented as mean ± SD (*n* = 3) at each fermentation time point. Asterisks (*, **, ***) indicate significant differences from Day 0 within the same assay (Welch’s *t*-test; unadjusted *p* < 0.05, 0.01, and 0.001, respectively; Šídák-adjusted *p*-values are provided in Table S4).

*L. rhamnosus*-fermented extracts were screened at two-day intervals for antioxidant (FRAP and DPPH), antidiabetic, anti-obesity, and antimicrobial activities over the ten days (Figure 2). Each activity is described in turn below.

#### 3.2.1. Antioxidant Activity

The antioxidant capacity of *L. rhamnosus*-fermented BSG extracts, assessed by both FRAP and DPPH assays, showed a similar temporal pattern, with maximum activity observed on Day 2 followed by a progressive decline through to Day 10 (Figure 2A,B). FRAP activity started at 1.0 mg TEAC/g BSG on Day 0, rose to its maximum of 1.24 mg TEAC/g BSG on Day 2, and decreased to its lowest value of 0.70 mg TEAC/g BSG on Day 10. Similarly, DPPH activity started at approximately 0.6 mg TEAC/g BSG on Day 0, peaked at 1.12 mg TEAC/g BSG on Day 2, and declined steadily to approximately 0.39 mg TEAC/g BSG by Day 10. However, the time-matched uninoculated control behaved differently. Its FRAP activity increased from 1.02 mg TEAC/g BSG on Day 0 to 1.42 mg TEAC/g BSG on Day 10, exceeding that of the fermented samples from Day 4 onwards (Figure S2A). In contrast, the DPPH activity of the control remained between 0.51 and 0.74 mg TEAC/g BSG throughout the incubation period. The Day 2 fermented samples therefore showed the clearest increase in DPPH activity relative to the control (Figure S2B).

The early peak in antioxidant activity observed on Day 2 aligns with the increased metabolic activity of *L. rhamnosus* during its exponential growth phase, as indicated by the simultaneous maximum in viable cell counts (Figure 1B). During this active growth period, LAB are known to generate diverse metabolites, including short-chain organic acids, exopolysaccharides, free radical-scavenging compounds, and bioactive peptides released through proteolysis, which have been reported to contribute to enhanced antioxidant capacity [6,33]. In addition, the decline in antioxidant activity observed after Day 2 in the fermented samples, but not in the uninoculated control, appears to be related to microbial fermentation rather than substrate incubation alone. It may reflect the consumption of phenolic compounds and other fermentable substrates by the growing culture, together with the degradation of labile antioxidant compounds under the increasingly acidic conditions generated during prolonged fermentation [33,34]. However, these mechanisms remain speculative, as organic acids, phenolic compounds, and peptides were not quantified in the present study.

#### 3.2.2. Antidiabetic Activity

α-Glucosidase inhibitory activity followed a different pattern from antioxidant activity (Figure 2C). On Day 0, the three replicates varied considerably, ranging from 0.12 to 1.71 mg AE/g BSG, with a mean of 0.67 mg AE/g BSG. The baseline was therefore poorly defined, and values at this level should be considered semi-quantitative. After fermentation began, activity increased to 2.81 mg AE/g BSG on Day 2 and then decreased to 1.86 mg AE/g BSG on Day 4. It subsequently increased again on Days 6 and 8, reaching the highest value of 3.22 mg AE/g BSG on Day 10. In contrast, the uninoculated control remained between 0.17 and 1.24 mg AE/g BSG throughout the ten-day period and showed no consistent trend (Figure S2C).

From Day 2 onwards, α-glucosidase inhibitory activity in every fermented extract was higher than the highest value observed in the uninoculated control. This indicates that the increased activity was generated during fermentation rather than simply released from the BSG during incubation. The increase can be associated with the growth of *L. rhamnosus*, which dominated the substrate during fermentation (Figure 1B). *L. rhamnosus* can produce proteases and carbohydrate-active enzymes that may liberate bioactive peptides and phenolic acids from the complex BSG matrix. These compounds are known to inhibit carbohydrate-metabolising enzymes such as α-glucosidase [6,35]. The progressive increase in activity up to Day 10, despite declining viable cell counts, may reflect ongoing enzymatic activity or the delayed accumulation of bioactive compounds after active growth had ceased. Cell-envelope proteinases of *Lactobacillus* species can remain functional independently of the producing cells, and prolonged fermentation has been associated with the continued accumulation of bioactive peptides [16,36]. However, neither proteolysis nor peptide release was measured in the present study; this remains an interpretation rather than a demonstrated mechanism. Future proteomic and metabolomic approaches would allow the responsible bioactive compounds to be identified and characterised.

#### 3.2.3. Anti-Obesity Activity

Anti-obesity activity showed the largest increase during incubation among all bioactivities assessed (Figure 2D). Similar to antidiabetic activity, the inoculated baseline exhibited no detectable activity on Day 0, while a substantial increase to approximately 4.3 mg OE/g BSG was observed on Day 2. Activity continued to rise through Days 4 and 6, reaching approximately 6.0 and 6.7 mg OE/g BSG, respectively, before a slight decrease on Day 8 (5.24 mg OE/g BSG), followed by an increase to the highest recorded value on Day 10 (6.9 mg OE/g BSG). The uninoculated control showed no lipase inhibition on Days 0 and 2. Activity then appeared, but remained between 1.59 and 2.68 mg OE/g BSG from Day 4 to Day 10, well below the values measured in the fermented extracts at the same time points (Figure S2D).

The very low anti-obesity activity at the inoculated baseline, followed by a steady increase during incubation, indicates that fermentation, in which *L. rhamnosus* was the dominant organism, contributed to the formation of lipase-inhibitory compounds. The appearance of lipase-inhibitory activity in the control from Day 4 indicates that part of this response arises from the substrate and its native microbiota, whereas the larger increase in the inoculated samples is attributable to the fermentation itself. These compounds may include fatty acid derivatives formed during fermentation, phenolics released from the cell wall matrix, and bioactive peptides produced through proteolysis [5,6]. The continuous increase in activity, with the highest value recorded on Day 10, suggests that anti-obesity compounds might continue to accumulate after viable bacterial counts had peaked and begun to decline. This trend is similar to the antidiabetic profile observed in this study. It also supports the view that enzymatic substrate breakdown can continue even after net microbial growth has stopped [37]. Taken together, the antidiabetic and anti-obesity results suggest that longer fermentation times with *L. rhamnosus* may be preferable when the aim is to maximise these specific health-related properties during BSG valorisation.

#### 3.2.4. Antimicrobial Activity

Antimicrobial activity of *L. rhamnosus*-fermented BSG extracts against the three indicator bacteria is summarised in Figure 2E as mean MBC (mg BSG/mL), with the inoculated Day 0 baseline used as the reference. No bactericidal effect was detected against either *S. aureus* or *E. coli* on Day 0. From Day 2 onward, consistent MBC values of 166.7 mg BSG/mL were observed against both organisms and remained unchanged through Day 10. *B. cereus* exhibited a distinct pattern: on Day 0, the inoculated baseline extract already achieved an MBC of 166.7 mg BSG/mL, suggesting that components active against this organism were present before incubation, possibly originating from the substrate itself. From Day 2 onward, however, the MBC against *B. cereus* decreased to 83.3 mg BSG/mL and remained at this lower value, indicating stronger bactericidal potency through the end of the fermentation period. MIC values were determined in parallel and were identical to the corresponding MBC values for all extracts and all three indicator bacteria. The MBC/MIC ratio was 1 in every case, indicating bactericidal rather than bacteriostatic activity [22]. The uninoculated control was tested in parallel at every time point. No bactericidal effect was detected against *S. aureus* or *E. coli* in the control on any day. Against *B. cereus*, the control retained an MBC of 166.7 to 333.3 mg BSG/mL throughout, with no improvement over time (Table S3).

Bactericidal activity against *S. aureus* and *E. coli* appeared only after inoculation. The uninoculated control did not develop it at any point during the same incubation. The compounds responsible were produced during fermentation, rather than being released from the substrate by incubation alone. This effect may result from several classes of metabolites acting in combination. Organic acids, in their undissociated form, can penetrate the bacterial cytoplasmic membrane and dissociate intracellularly, lowering cytoplasmic pH and disrupting the proton motive force [38]. Phenolic compounds may alter membrane permeability and compromise cell integrity through interactions with the cell membrane and intracellular enzymes [39], while proteolytically derived peptides and bacteriocin-like substances may interfere with cell wall synthesis or essential intracellular processes [40,41]. Although these metabolites can act against both Gram-positive and Gram-negative bacteria, Gram-positive organisms are often more susceptible owing to the absence of an outer membrane barrier. None of these metabolite classes were measured here; thus, their relative contribution to the observed activity cannot be determined.

*B. cereus* was a notable exception, as susceptibility was already observed in the Day 0 baseline extract. The uninoculated control showed the same activity against this organism and did not improve over the incubation period. That activity therefore likely originated from compounds already present in BSG. Fermentation further improved this activity, as shown by the two-fold decrease in MBC values from Day 2 onwards. The stable MBC values from Days 2 to 10 for all three bacteria indicate that the main antimicrobial metabolites reached effective concentrations during the early growth phase. These concentrations were then maintained throughout fermentation, even as viable cell counts declined.

### 3.3. Global Bioactivity Score of L. rhamnosus-Fermented BSG

**Figure 3:**
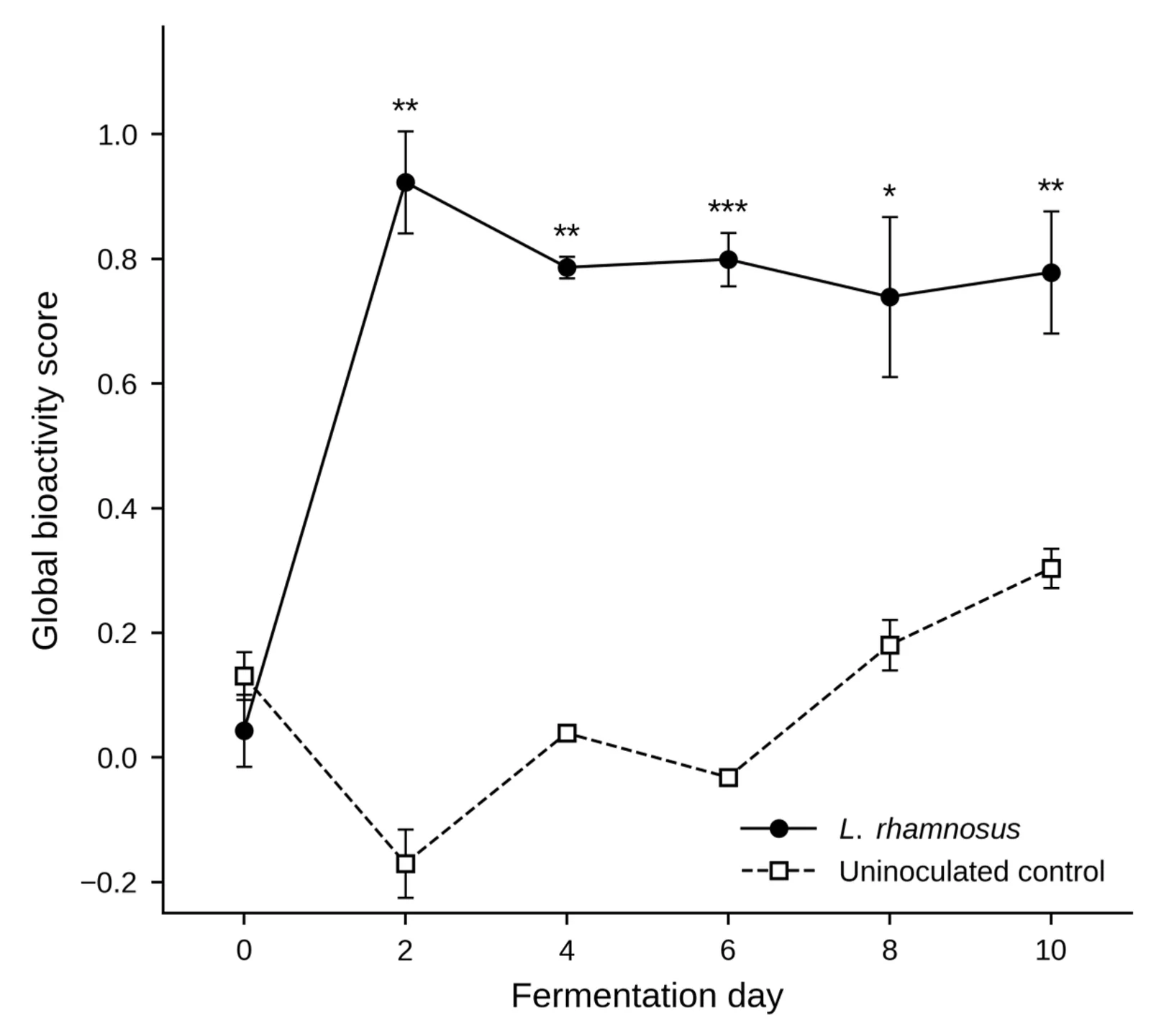
Normalized global bioactivity score of *Lacticaseibacillus rhamnosus* (*L. rhamnosus*, ●)-fermented brewer’s spent grain (BSG) over a 10-day fermentation period and uninoculated BSG (control, □). The global bioactivity score combines antioxidant (FRAP and DPPH), antidiabetic, anti-obesity, and antimicrobial activities and was normalized to a 0–1 scale using the minimum and maximum values observed in the *L. rhamnosus* fermentation time course; consequently, projected control values may fall outside this range. Data are presented as mean ± SD from three independent biological replicates for *L. rhamnosus* (*n* = 3) and two independent biological replicates for the control (*n* = 2). Asterisks indicate significant differences from Day 0 for *L. rhamnosus* (* *p* < 0.05; ** *p* < 0.01; *** *p* < 0.001). No significance testing was applied to the control.

The four bioassays were measured in different units and on different scales and thus could not be directly compared or combined. Each assay was standardised and averaged, with equal weight given to antioxidant, antidiabetic, anti-obesity, and antimicrobial activity, to give a single global bioactivity score for each fermentation day (Figure 3).

The inoculated baseline at Day 0 showed a low score of approximately 0.04, indicating limited overall bioactive potential at the start of incubation. The score increased sharply from 0.04 on Day 0 to 0.92 on Day 2. After reaching its maximum on Day 2, the score remained at 0.79 on Day 4 and 0.80 on Day 6, declined slightly to 0.74 on Day 8, and recovered to 0.78 on Day 10. Significant differences from Day 0 were observed at all later time points after Šídák adjustment for five comparisons, with adjusted *p* < 0.001 on Day 6, *p* < 0.01 on Days 2, 4 and 10, and *p* < 0.05 on Day 8.

A sensitivity analysis (Table S2A), in which the global bioactivity score was recalculated after removing each of the four bioactivity domains in turn, showed that Day 2 remained the highest-scoring time point when the antidiabetic, anti-obesity, or antimicrobial domain was excluded. However, when the antioxidant domain was excluded, the highest-scoring time point shifted to Day 10. The identification of Day 2 as the highest-scoring time point therefore depended on the inclusion of antioxidant activity, consistent with the opposing temporal trends observed among the individual bioactivities.

The uninoculated control remained relatively stable over the 10-day incubation period, whereas the fermented samples exceeded the control from Day 2 onwards. These findings suggest that the increase in global bioactivity was driven primarily by microbial fermentation rather than by substrate incubation alone. This comparison is descriptive, since the control was prepared in duplicate and no statistical test was applied to it.

From a bioprocessing perspective, Day 2 can be considered the highest-scoring endpoint and hence the preferred fermentation time when maximising overall bioactivity is the primary objective. However, the relatively narrow range of scores observed from Days 4 to 10 indicates that *L. rhamnosus* fermentation maintains a stable multifunctional bioactive profile beyond the early growth peak. This may provide operational flexibility when longer fermentation times are required.

### 3.4. Antimicrobial Activity on Food Matrices

To assess the practical relevance of the antimicrobial activity reported in Section 3.2.4, the Day 2 *L. rhamnosus*-fermented BSG extract was selected on the basis of its highest global bioactivity score (Section 3.3). This experiment was designed as a proof of concept to determine whether activity observed in broth was retained in a food matrix, rather than as a time-course study; therefore, a single representative time point was used.

The extract was tested against *E. coli*, *B. cereus*, and *S. aureus* in two food-simulating matrices: a wheat-based bread medium and a dairy-based skim milk medium. Bactericidal activity was generally stronger and more consistent in the bread medium than in the milk medium (Table 1).

In the bread medium, bactericidal activity was observed against all three bacteria at 333.3, 166.7, and 83.3 mg BSG/mL. At the lowest concentration tested (41.7 mg BSG/mL), no bactericidal effect was observed against *E. coli*, whereas partial inhibition was recorded for *B. cereus* and *S. aureus*. In the milk medium, bactericidal activity was considerably weaker, with all three bacteria showing bactericidal inhibition only at the highest concentration tested (333.3 mg BSG/mL) and no inhibitory effect detected at lower concentrations.

The bactericidal activity observed in the bread medium was consistent with our previous findings [12], in which *L. plantarum*-fermented BSG extracts showed stronger antimicrobial efficacy in wheat-based than in milk-based media. This difference may be attributed to the composition of the bread matrix. The bread medium was produced through enzymatic hydrolysis using proteases and α-amylases, which break down complex macromolecules into smaller, more accessible molecules. As a result, the matrix may have a reduced capacity to non-specifically bind antimicrobial metabolites, allowing organic acids, phenolic compounds, and bioactive peptides to remain bioavailable and interact more effectively with the bacterial cell membrane [42,43]. The reduced activity observed in the milk medium appears to reflect the physicochemical properties of the dairy matrix rather than the absence of intrinsic antimicrobial potential. Skim milk contains casein micelles and whey proteins, which can interact with phenolic compounds, organic acids, and peptide-based antimicrobials through hydrophobic interactions and hydrogen bonding. These interactions may reduce the concentration of free bioactive compounds available to act on bacterial cells [44,45]. Nevertheless, this interpretation is based on evidence from the literature, as the chemical composition of the extracts was not determined in the present study.

The partial inhibition of *B. cereus* and *S. aureus* at 41.7 mg BSG/mL further suggests a dose-dependent antimicrobial response. Although colonies were still recovered at this concentration, their numbers were reduced relative to the growth control, indicating that the minimum bactericidal concentration for these Gram-positive bacteria may be close to the tested range. In contrast, *E. coli* was not inhibited at this concentration. This difference is likely due to its outer membrane, which is rich in lipopolysaccharides and acts as a permeability barrier. This may restrict the entry of phenolic compounds, organic acids, and antimicrobial peptides, thereby requiring higher concentrations to achieve bactericidal activity against Gram-negative bacteria [6,40].

Collectively, these results indicate that *L. rhamnosus*-fermented BSG extracts retain measurable antimicrobial activity in food-relevant matrices. However, their efficacy is strongly influenced by matrix composition. The stronger performance observed in the bread medium suggests that cereal-based products may represent a more promising application platform than dairy systems at equivalent extract concentrations.

### 3.5. Bioactivity Screening in L. fermentum Fermented BSG

As shown in Section 3.1, *L. fermentum* fermentations were affected by polymicrobial contamination from Day 4 onwards. Thus, the bioactivity results presented in this section should not be interpreted as the specific metabolic output of *L. fermentum*. Instead, they reflect the combined activity of the mixed microbial community that developed during fermentation. These data are reported as exploratory observations and cannot be used to define the intrinsic bioactive potential of *L. fermentum* on BSG under controlled fermentation conditions.

**Figure 4:**
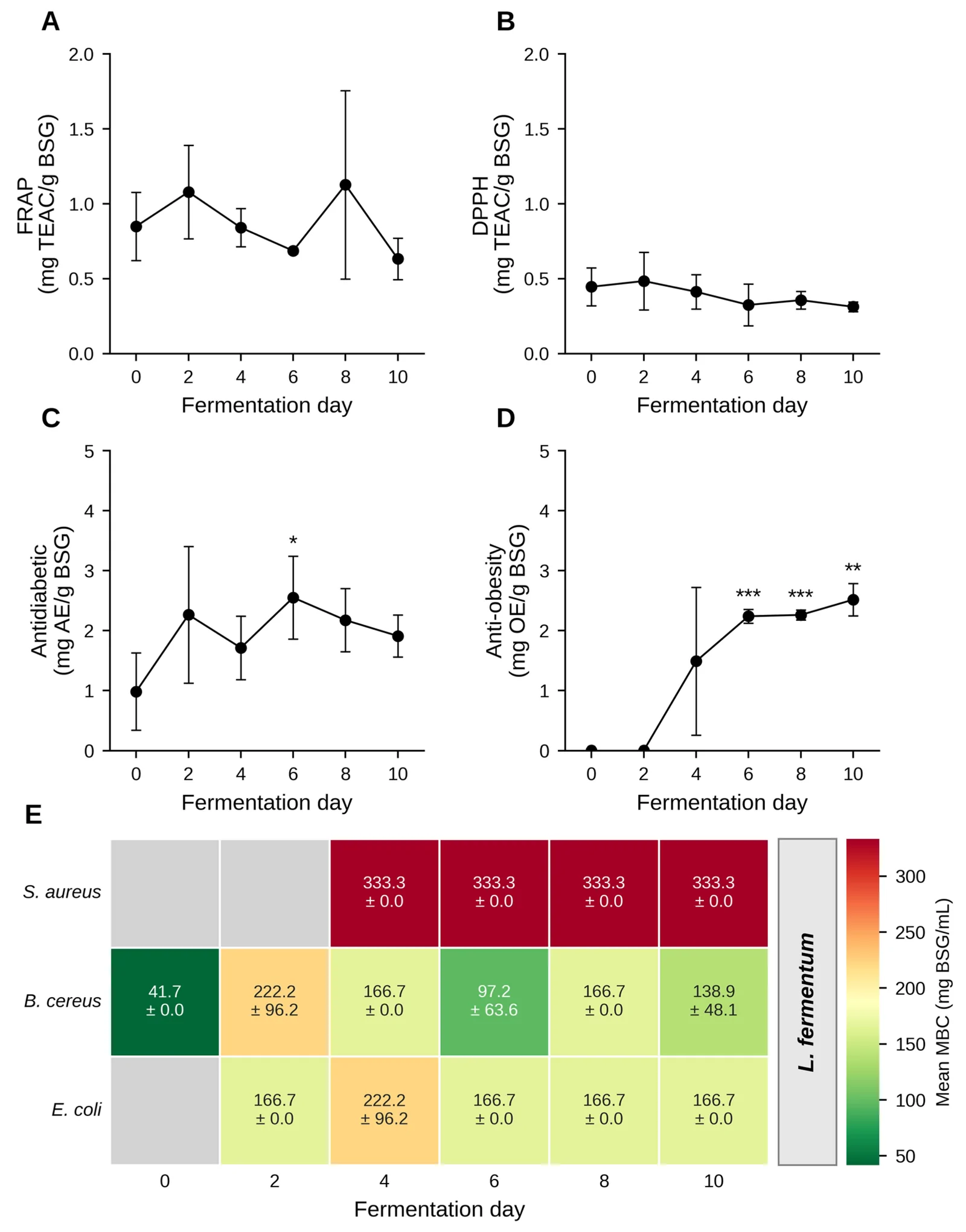
Time-course changes in the bioactivity of *L. fermentum*-fermented brewer’s spent grain (BSG) extracts over a 10-day period. (**A**,**B**) Antioxidant activity measured by FRAP and DPPH, respectively, expressed as mg TEAC/g BSG. (**C**) Antidiabetic activity, expressed as mg AE/g BSG. (**D**) Anti-obesity activity, expressed as mg OE/g BSG. (**E**) Antimicrobial activity expressed as the mean minimum bactericidal concentration (MBC, mg BSG/mL) against *Staphylococcus aureus* (*S. aureus*), *Bacillus cereus* (*B. cereus*), and *Escherichia coli* (*E. coli*) across the fermentation period; lower MBC values indicate stronger antibacterial activity. Grey cells indicate no bactericidal effect within the tested concentration range. Data are presented as mean ± SD (*n* = 3) at each fermentation time point. Where one replicate showed no bactericidal effect, the mean and SD were calculated from the two quantifiable replicates (*S. aureus* on Day 8, *B. cereus* on Day 0, and *E. coli* on Days 2 and 6). Asterisks (*, **, ***) indicate significant differences from Day 0 within the same assay (Welch’s *t*-test; unadjusted *p* < 0.05, 0.01, and 0.001, respectively; Šídák-adjusted *p*-values are provided in Table S4).

The antioxidant activity of *L. fermentum*-fermented BSG extracts, measured using FRAP and DPPH assays, showed different patterns over the fermentation period, with FRAP values fluctuating while DPPH activity remained relatively stable (Figure 4A,B). The fluctuating FRAP values, particularly the increase on Day 8 and the high variability between replicates, indicate a less consistent antioxidant response than that observed for *L. rhamnosus* (Figure 2A). This variability may reflect changes in the mixed microbial environment during fermentation. Hence, the results do not provide strong evidence that *L. fermentum* fermentation enhanced antioxidant capacity.

Antidiabetic activity also showed high variability between replicates throughout fermentation (Figure 4C). Activity increased from approximately 1.0 mg AE/g BSG on Day 0 to ~2.3 mg AE/g BSG on Day 2, peaked at ~2.55 mg AE/g BSG on Day 6, and then declined slightly. The fluctuating trend and large error bars prevent any clear conclusion about fermentation effects, unlike the more consistent response observed with *L. rhamnosus.* However, anti-obesity activity followed a distinct temporal pattern (Figure 4D). Anti-obesity activity was absent on Days 0 and 2 but emerged on Day 4, reaching ~1.5 mg OE/g BSG and stabilising at ~2.2–2.55 mg OE/g BSG from Days 6–10. As this increase occurred after contamination began, it likely reflects the activity of contaminating microorganisms rather than *L. fermentum* alone. Nevertheless, the result suggests that BSG may support the production of lipase-inhibitory compounds by mixed microbial communities.

The antimicrobial activity of *L. fermentum* extracts (Figure 4E) was generally weak, variable, and less consistent than that of *L. rhamnosus*. Against *S. aureus*, bactericidal activity was detected from Day 4 onwards at 333.3 mg BSG/mL. On Day 8, activity was detected in two of the three replicates, while no effect was detected in the third. For *B. cereus*, bactericidal activity at Day 0 was detected at 41.7 mg BSG/mL in two of the three replicates, whereas no effect was observed in the third. From Days 2 to 10, mean MBC values ranged from 97.2 to 222.2 mg BSG/mL. The lower susceptibility of *B. cereus* after fermentation is unlikely to be attributable to the fermentation process itself and may instead reflect interactions between metabolites produced by contaminating microorganisms and antimicrobial compounds already present in the substrate at Day 0. Against *E. coli*, no bactericidal activity was detected on Day 0. From Days 2 to 10, mean MBC values ranged from 166.7 to 222.2 mg BSG/mL; on Days 2 and 6, activity was detected in two of the three replicates but not in the third. Overall, the large standard deviations observed across fermentation time points and indicator bacteria suggest considerable instability within the fermentation system. This variability likely arose from differences in the composition and abundance of contaminating microorganisms among replicates rather than from controlled biological variation [46]. MIC values were determined in parallel and were identical to the corresponding MBC values for all quantifiable extracts and indicator bacteria. Accordingly, the MBC/MIC ratio was 1 in all quantifiable cases, consistent with a bactericidal effect rather than a bacteriostatic effect [22].

Comparison with the uninoculated control showed that the effects of *L. fermentum* inoculation were limited. Antioxidant, antidiabetic, and anti-obesity activities were generally similar to or lower than those observed in the uninoculated control (Figure S2). The main exception was antimicrobial activity, as the uninoculated control showed no activity against *S. aureus* or *E. coli*. Hence, while *L. fermentum* inoculation generated some antimicrobial activity, it provided little additional improvement in the other measured bioactivities beyond that achieved through spontaneous fermentation. This limited enhancement prompted the subsequent heat-pretreatment experiments described in Section 3.6, which investigated whether modifying the substrate could improve fermentation performance and functional outcomes.

In summary, the *L. fermentum* results highlight the importance of microbial control for reliable bioactivity assessment. Failure of *L. fermentum* to establish itself as the predominant microorganism in untreated BSG resulted in substantial contamination and reduced confidence in attributing observed bioactivities to the target strain.

### 3.6. Impact of BSG Heat Pretreatments on L. fermentum Fermentation

#### 3.6.1. pH and Cell Growth

**Figure 5:**
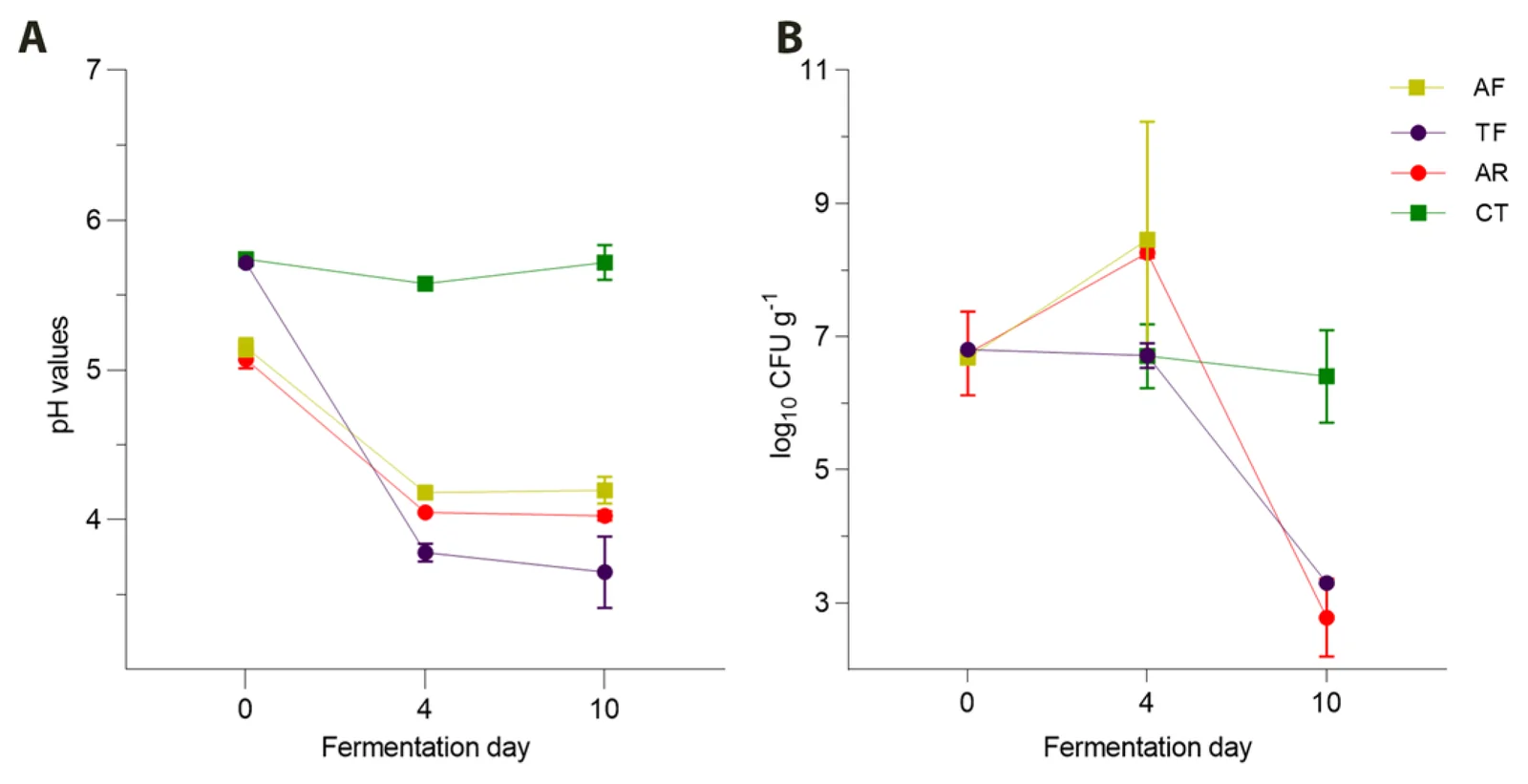
Fermentation dynamics of heat-pretreated brewer’s spent grain (BSG). Changes in pH (**A**) and viable cell counts (**B**) were monitored at fermentation Days 0, 4, and 10 in four fermentations: autoclaved BSG inoculated with *L. fermentum* (AF), two-step heat-treated BSG inoculated with *L. fermentum* (TF), autoclaved BSG inoculated with *L. rhamnosus* as a positive control (AR), and uninoculated two-step heat-treated BSG as a control (CT). Data are presented as mean ± SD (*n* = 3), except for TF on Day 10, where only one of three replicates was quantifiable and a single observation without an SD. No quantifiable colony-count data were available for AF on Day 10. CT produced zero colonies on Day 0.

To investigate the contamination observed during *L. fermentum* fermentation, raw BSG was subjected to autoclaving or a two-step heat treatment. Four fermentations were evaluated: autoclaved BSG inoculated with *L. fermentum* (AF), two-step heat-treated BSG inoculated with *L. fermentum* (TF), autoclaved BSG inoculated with *L. rhamnosus* (AR), and an uninoculated two-step heat-treated BSG (CT). Full pH and microbial growth data are presented in Figure 5.

Both AR and AF acidified rapidly, reaching pH 4.05 and 4.18, respectively, on Day 4, with no further decrease by Day 10. TF reached pH 3.78 on Day 4 and 3.65 on Day 10, whereas CT remained between pH 5.58 and 5.74 throughout the fermentation period (Figure 5A).

In AF, viable counts peaked at 8.45 log_10_ CFU g^−1^ on Day 4 before declining below the detection limit by Day 10 (Figure 5B). In TF, viable counts did not increase above the initial level and fell below the detection limit on Day 10, indicating no net proliferation. Notably, in AR, *L. rhamnosus* reached 8.26 log_10_ CFU g^−1^ on Day 4, comparable to its growth in untreated BSG (Figure 1B). Together with the growth observed in AF, this indicates that autoclaving did not impair the ability of BSG to support initial LAB proliferation. Although no colonies were detected in CT on Day 0, heavy contamination developed by Day 4 (Figure S4), reaching 6.70 log_10_ CFU g^−1^. The stable pH of CT suggests that the contaminating microorganisms produced little or no acid.

Overall, neither heat pretreatment enabled successful *L. fermentum* fermentation. Although autoclaving supported initial growth, viable cells were no longer detectable by Day 10. In contrast, the two-step heat treatment neither supported *L. fermentum* proliferation nor prevented microbial growth in the uninoculated CT control. Hence, heat-based pretreatment alone appears insufficient to support successful *L. fermentum* fermentation of BSG. However, the heat pretreatment experiment included only three sampling points (Days 0, 4, and 10), compared with six in the main fermentation experiment, reducing kinetic resolution and limiting direct comparison of microbial growth and acidification patterns. Consequently, the timing and causes of the decline in *L. fermentum* viability, particularly in AF, could not be determined with certainty. Future studies should investigate alternative strategies, including enzymatic pretreatment, nutrient supplementation, and controlled strain adaptation [47,48,49,50].

#### 3.6.2. Bioactivities of Heat-Pretreated BSG

To evaluate whether heat pretreatment influenced the bioactivity of *L. fermentum* fermented BSG, antioxidant and enzyme-inhibitory activities were compared between AF and TF samples (Figure S5). Heat pretreatment had limited effects on antioxidant and antidiabetic activities but markedly enhanced anti-obesity activity. FRAP activity decreased significantly in AF from 0.95 to 0.66 mg TEAC/g BSG between Days 0 and 10 (*p* = 0.037), whereas no significant change was observed in TF (Figure S5A). DPPH radical scavenging and antidiabetic activities remained relatively stable throughout fermentation in both treatments (Figure S5B,C)). In contrast, anti-obesity activity increased significantly during fermentation, reaching 2.63 and 7.25 mg OE/g BSG in AF and TF, respectively, on Day 10 (Figure S5D). Notably, the anti-obesity activity observed in TF on Day 10 was slightly higher than the maximum value recorded for *L. rhamnosus*-fermented BSG (Figure 2D).

These results suggest that heat pretreatment primarily affected lipase-inhibitory activity rather than antioxidant or antidiabetic properties. Unlike untreated BSG, which showed no anti-obesity activity prior to *L. fermentum* fermentation (Figure 4D), both heat-treated substrates exhibited measurable activity at Day 0, indicating that heat treatment itself contributed to lipase inhibition. Fermentation further enhanced this activity, with a greater increase observed in TF. This finding suggests that substrate modifications induced by heat treatment, rather than microbial growth alone, were responsible for the enhanced activity. As BSG proteins are known precursors of pancreatic lipase-inhibitory peptides [51], and heat treatment can alter protein structure and susceptibility to proteolysis [52], the substantially higher anti-obesity activity in TF may reflect differences in protein modification during pretreatment. This hypothesis was further investigated through proteomic analysis.

#### 3.6.3. BSG Protein Composition

Autoclaving removed the competing microorganisms, yet *L. fermentum* still failed to establish a successful fermentation (Section 3.6.1), while the two pretreatments produced markedly different anti-obesity activities (Section 3.6.2). Both observations point to the substrate rather than the microorganism. Because LAB rely on substrate proteins as a nitrogen source, and BSG proteins have been reported to be a source of lipase-inhibitory peptides [51], FASP-based proteomic analysis was performed on uninoculated raw, two-step heat-treated, and autoclaved BSG to determine how each treatment affected the protein composition.

**Figure 6:**
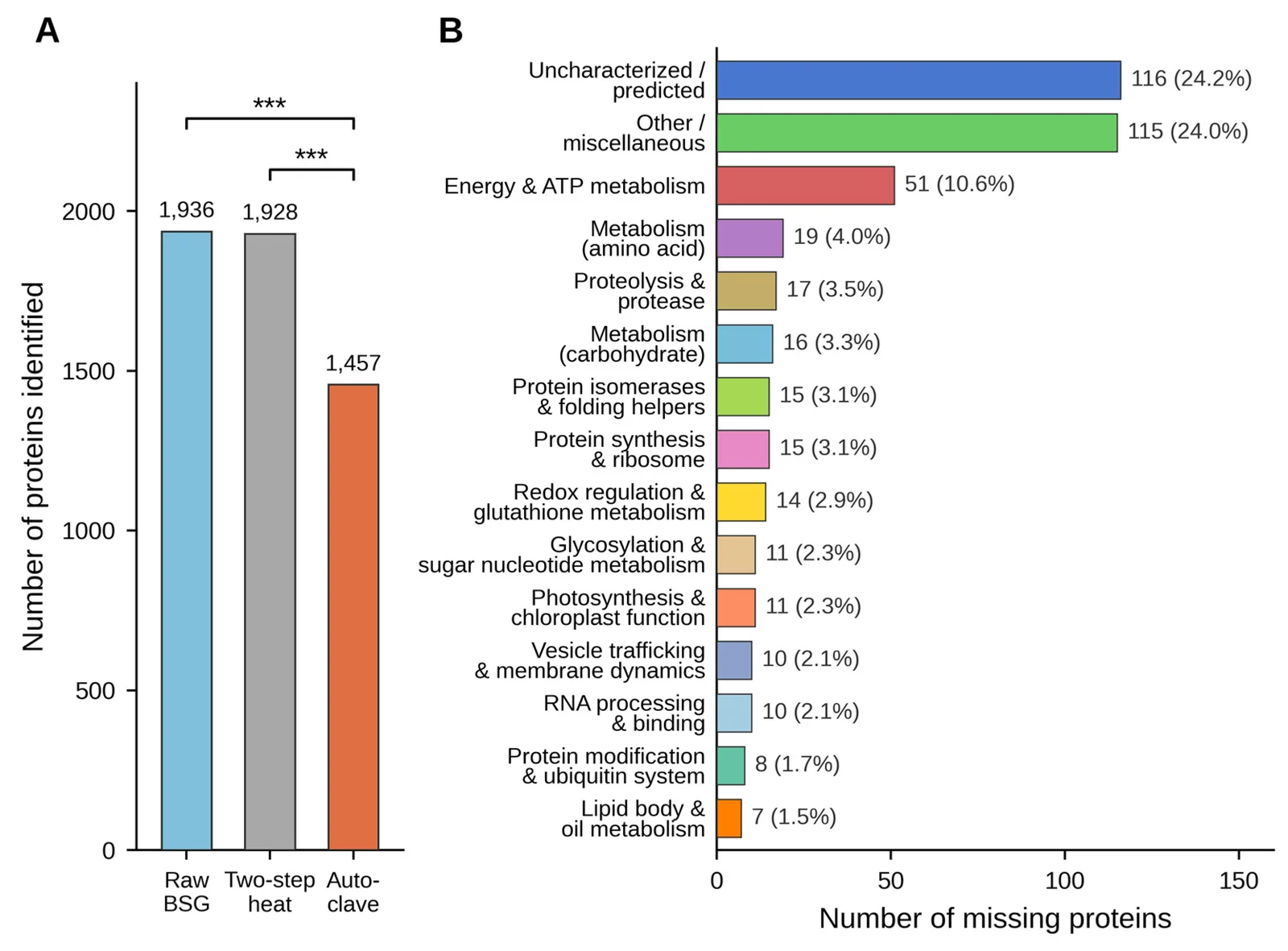
Proteomic characterisation of brewer’s spent grain (BSG) subjected to different heat pretreatments. (**A**) Total number of proteins identified by LC-MS/MS in raw BSG, two-step heat-treated BSG, and autoclaved BSG. Asterisks (***) indicate significant differences between raw and autoclaved BSG and between two-step heat-treated and autoclaved BSG, based on two-sided Welch’s *t*-tests with Šídák adjustment for three pairwise comparisons (adjusted *p* < 0.001). (**B**) Functional classification of the 479 proteins identified in raw BSG but not detected after autoclaving, showing the 15 most abundant functional categories in descending order of frequency.

Figure 6 shows that, based on the number of detected proteins, two-step heat treatment had minimal impact on the BSG proteome. A total of 1928 proteins were identified after two-step heat treatment, compared with 1936 proteins in the raw BSG samples, representing a loss of only eight proteins (0.4%), which was not statistically significant (*p* > 0.05; Figure 6A). In contrast, autoclaving resulted in substantial protein loss. Only 1457 proteins remained detectable after autoclaving, corresponding to a significant reduction of 479 proteins (24.7%) relative to the raw BSG (*p* < 0.001). Among these 479 proteins, many were associated with biologically important functions, including energy and ATP metabolism (*n* = 51, 10.6%), amino acid metabolism (*n* = 19, 4.0%), proteolytic enzymes (*n* = 17, 3.5%), protein synthesis and ribosomal components (*n* = 15, 3.1%), redox regulation (*n* = 14, 2.9%), and RNA processing and binding (*n* = 10, 2.1%) (Figure 6B).

The proteomic differences may help explain the contrasting anti-obesity activities observed for AF and TF (Section 3.6.2, Figure S5D). The two-step heat treatment preserved a protein profile that was more similar to that of raw BSG, which may have supported the greater lipase-inhibitory activity observed following TF fermentation. In contrast, autoclaving reduced the number of detectable proteins, which may have limited the development of lipase-inhibitory activity in AF. Because BSG proteins can serve as precursors for lipase-inhibitory peptides, these results suggest that preservation of BSG proteins and their subsequent breakdown into bioactive peptides during fermentation may have contributed to the observed activity. Moreover, many of the reduced proteins were associated with important metabolic functions. This change may have limited the availability of protein-derived substrates necessary to support continued bacterial growth [53]. The absence of further acidification after Day 4, together with the lack of detectable viable colonies at the end of fermentation in AF (Figure 5), suggests that conditions may have become unfavourable for *L. fermentum* growth and survival, potentially due to limited nutrient availability.

## 4. Conclusions

*L. rhamnosus* successfully fermented raw BSG and increased antioxidant, antidiabetic, anti-obesity, and antimicrobial activities. Antioxidant activity peaked on Day 2, whereas antidiabetic and anti-obesity activities reached their highest levels on Day 10, indicating that different bioactive properties developed at different stages of fermentation. The global bioactivity score identified Day 2 as the highest-scoring endpoint under the conditions tested, while bactericidal activity in a bread-based matrix highlighted the potential application of *L. rhamnosus*-fermented BSG as a natural preservative ingredient in cereal-based foods.

In contrast, *L. fermentum* failed to establish a successful fermentation in BSG, even after heat pretreatment. Although autoclaving improved short-term proliferation, viable cells were no longer detectable by Day 10, and the two-step heat treatment neither increased total viable counts nor provided effective microbial control. Proteomic analysis showed that autoclaving substantially altered the detectable protein profile of BSG, which may have contributed to the lower lipase-inhibitory activity observed relative to the two-step heat-treated substrate. However, as bioactivity was not assessed in the autoclaved *L. rhamnosus* group, which served only as a growth control, the broader impact of autoclaving on bioactivity in otherwise successful BSG fermentations remains unclear and warrants further investigation. 

These findings demonstrate that successful BSG valorisation depends not only on microbial selection but also on strain-substrate compatibility and processing conditions. This study highlights the value of combining bioactivity profiling, food-model validation, and proteomic analysis to evaluate fermentation outcomes, and demonstrates that well-characterised unsuccessful fermentations can provide useful insights for process optimisation. Future work should focus on identifying the bioactive compounds responsible for the observed activities using proteomic and metabolomic approaches, and on exploring alternative strategies, such as enzymatic pretreatment, nutrient supplementation, or co-culture approaches, to improve fermentation performance.

## Figures and Tables

**Table 1:** Antibacterial activity of fermented brewer’s spent grain (BSG) extracts at different concentrations, expressed as BSG equivalents (mg fermented BSG per mL of assay medium), against foodborne pathogens in wheat and dairy-based media. “+” indicates a bactericidal effect (no colonies recovered on subculture); “−” indicates no bactericidal effect; and “±” indicates partial inhibition (a reduction in colony numbers relative to the growth control, with colonies still recovered). The growth control consisted of inoculated medium without extract.

Food Media	Bacteria	Concentration (mg BSG/mL)				Control
		333.3	166.7	83.3	41.7	
Bread	*E. coli*	+	+	+	−	FG
	*B. cereus*	+	+	+	±	FG
	*S. aureus*	+	+	+	±	LG
Milk	*E. coli*	+	−	−	−	FG
	*B. cereus*	+	−	−	−	FG
	*S. aureus*	+	−	−	−	FG

**Notes:** FG (full growth) showed CFU counts of 4.8 × 10^8^ (*E. coli*), 1.21 × 10^7^ (*B. cereus*), and 1.73 × 10^8^ (*S. aureus*) CFU/mL, while LG (low growth) exhibited 3.3 × 10^3^ CFU/mL for *S. aureus*.

## Data Availability

The original contributions presented in the study are included in the article/Supplementary Material, further inquiries can be directed to the corresponding author/s.

## Supplementary Materials

The following supporting information can be downloaded at: https://www.mdpi.com/article/10.3390/foods15203715/s1. **Figure S1**. Time-course changes in (A) pH and (B) viable cell counts of uninoculated brewer’s spent grain (control samples) during a 10-day incubation period. Data are presented as mean ± SD (*n* = 2), with viable cell counts expressed as log_10_ CFU g^−1^. The open triangle at Day 0 denotes a confirmed non-detect (below the detection limit of 2 log_10_ CFU g^−1^) and is plotted at a fixed low position; **Figure S2**. Time-course changes in (A,B) antioxidant activity measured by the FRAP and DPPH assays, respectively, (C) antidiabetic activity, and (D) anti-obesity activity of uninoculated brewer’s spent grain (BSG; control samples) during a 10-day incubation period. Data are presented as mean ± SD (*n* = 2). Activities are expressed as mg TEAC/g BSG (A-B), mg AE/g BSG (C), and mg OE/g BSG (D). Anti-obesity activity was not detected on Days 0 and 2 and is plotted as zero; **Figure S3**. Evidence of contamination during the fermentation of brewer’s spent grain with *Limosilactobacillus fermentum* on Day 4. (A) Visible fungal growth in the fermentation jar. (B) Morphologically distinct colonies observed on De Man, Rogosa and Sharpe (MRS) agar following plating of the fermentation sample. Both images were captured on Day 4 of fermentation; **Figure S4**. Heavy contamination observed on Day 4 in uninoculated, two-step heat-treated brewer’s spent grain (control samples) during the experiments described in Section 3.6; **Figure S5**. Bioactivity of heat-pretreated brewer’s spent grain (BSG) inoculated with *Limosilactobacillus fermentum* during a 10-day fermentation period. (A,B) Antioxidant activity measured by the FRAP and DPPH assays, respectively, expressed as mg TEAC/g BSG. (C) Antidiabetic activity, expressed as mg AE/g BSG. (D) Anti-obesity activity, expressed as mg OE/g BSG. AF and TF denote autoclaved and two-step heat-treated BSG, respectively. Data are presented as mean ± SD from three independent fermentation replicates (*n* = 3). Asterisks indicate significant differences relative to Day 0 within the same treatment and assay (Welch’s two-sample *t*-test; * *p *< 0.05, ** *p *< 0.01, *** *p *< 0.001); **Table S1**. Raw values and z-scores for each bioactivity domain, together with the global bioactivity score, by sample (Day × replicate) for *L. rhamnosus* only (*n* = 18); **Table S2A**. Leave-one-domain-out sensitivity analysis: mean global bioactivity score for each fermentation day and the resulting highest-scoring day when individual bioactivity domains were removed in turn (*L. rhamnosus*); **Table S2B**. MBC no-effect imputation sensitivity analysis: mean global bioactivity score for each fermentation day under different substituted values for "-" (no bactericidal effect) MBC results (*L. rhamnosus*). Because each MBC endpoint took only two distinct values across the dataset, z-standardisation removed the influence of the imputed magnitude; consequently, the global bioactivity score was identical for all substitutions tested; **Table S3**. Minimum bactericidal concentrations (MBCs) of the time-matched uninoculated brewer’s spent grain (BSG) control against three foodborne bacteria during a 10-day incubation period (*n* = 2); **Table S4**. Comparisons between Day 0 and each subsequent fermentation day for every individual bioassay shown in Figure 2 and Figure 4, reported as unadjusted and Šídák-adjusted *p*-values. Two-sided Welch’s two-sample *t*-tests were performed with *n* = 3 per group. The Šídák adjustment was applied across the five comparisons made within each assay and treatment (*p*adjusted = 1 − (1 − *p*)^5^). Asterisks in Figure 2 and Figure 4 correspond to the unadjusted *p*-values. Significance codes: *** *p* < 0.001, ** *p* < 0.01, * *p* < 0.05, ns = not significant. Rows in which the significance classification changed after adjustment are shaded.
