# Supplementary material for: Brewer’s Spent Grain as a Substrate for Lactic Acid Fermentation: Contrasting Performance of Lacticaseibacillus rhamnosus and Limosilactobacillus fermentum
Source: Foods. 2026 Oct 19;15(20):3715. doi: 10.3390/foods15203715 (PMC13704212; doi:10.3390/foods15203715)
Supplement: Supplementary file 1 [file foods-15-03715-s001.zip › Supplementary figures.pdf]

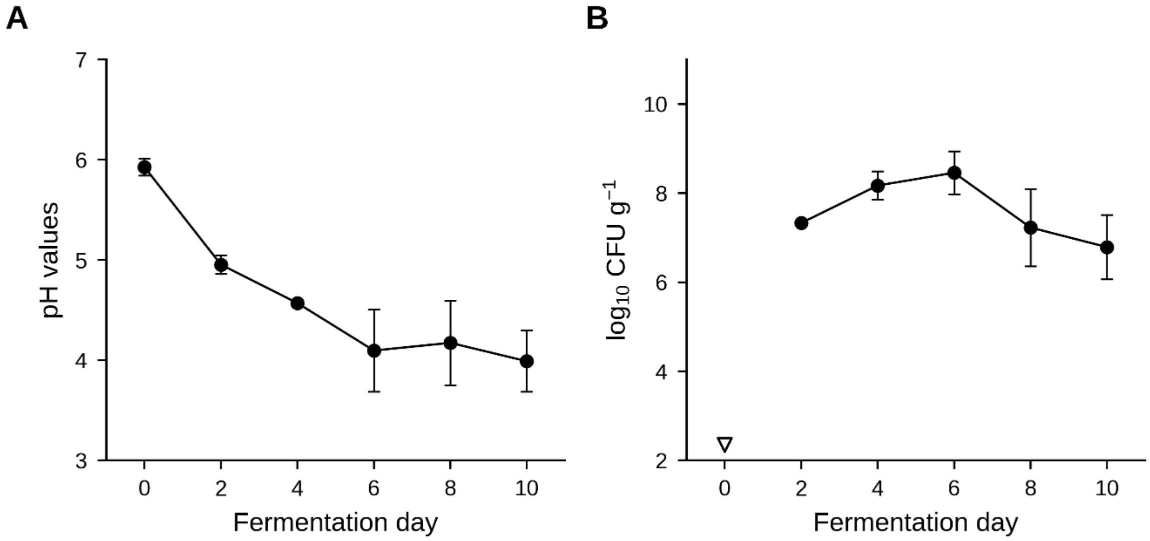

Figure S1. Time-course changes in (A) pH and (B) viable cell counts of uninoculated brewer's spent grain (control samples) during a 10-day incubation period. Data are presented as mean ± SD (n = 2), with viable cell counts expressed as $\log_{10}$ CFU g^{-1}. The open triangle at Day 0 denotes a confirmed non-detect (below the detection limit of 2 $\log_{10}$ CFU g^{-1}) and is plotted at a fixed low position.

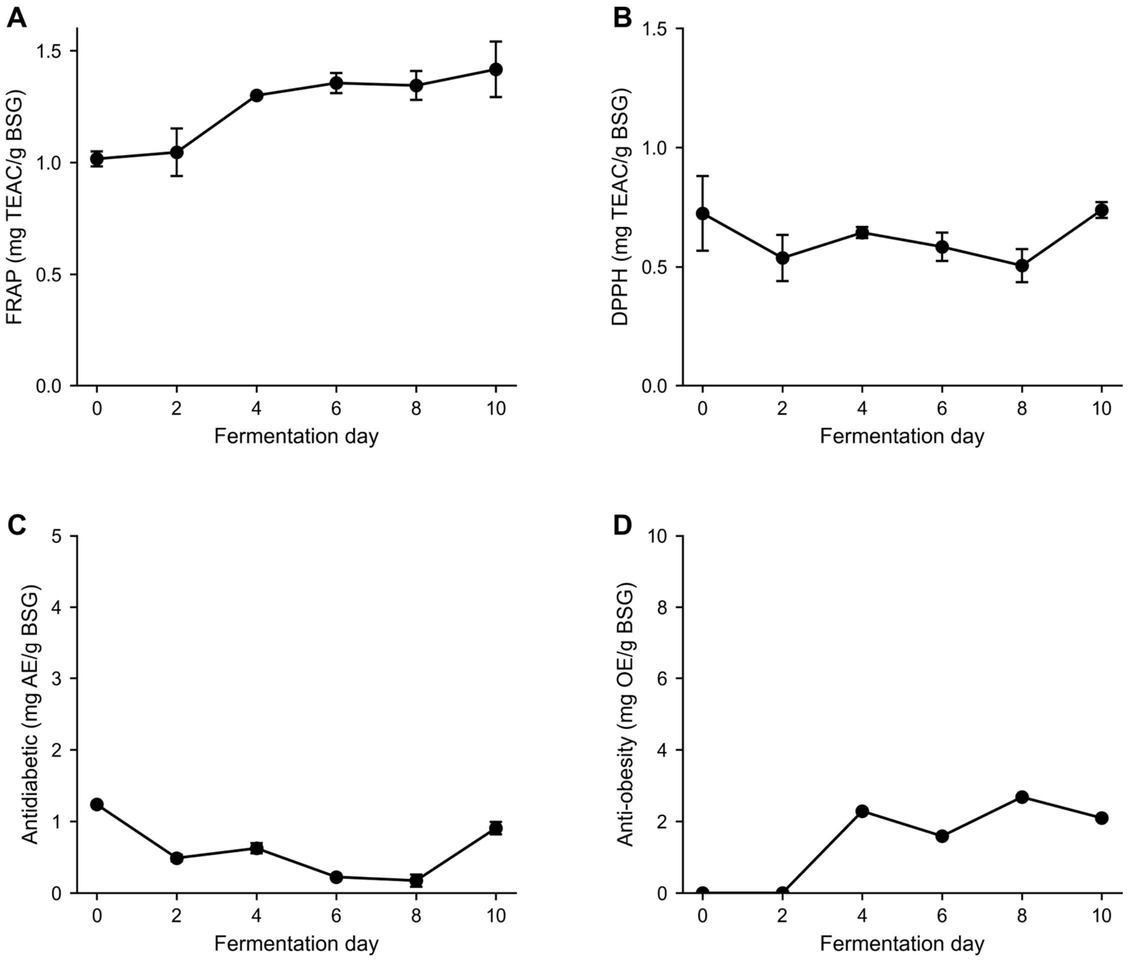

Figure S2. Time-course changes in (A-B) antioxidant activity measured by the FRAP and DPPH assays, respectively, (C) antidiabetic activity, and (D) anti-obesity activity of uninoculated brewer's spent grain (BSG; control samples) during a 10-day incubation period. Data are presented as mean ± SD (n = 2). Activities are expressed as mg TEAC/g BSG (A-B), mg AE/g BSG (C), and mg OE/g BSG (D). Anti-obesity activity was not detected on Days 0 and 2 and is plotted as zero.

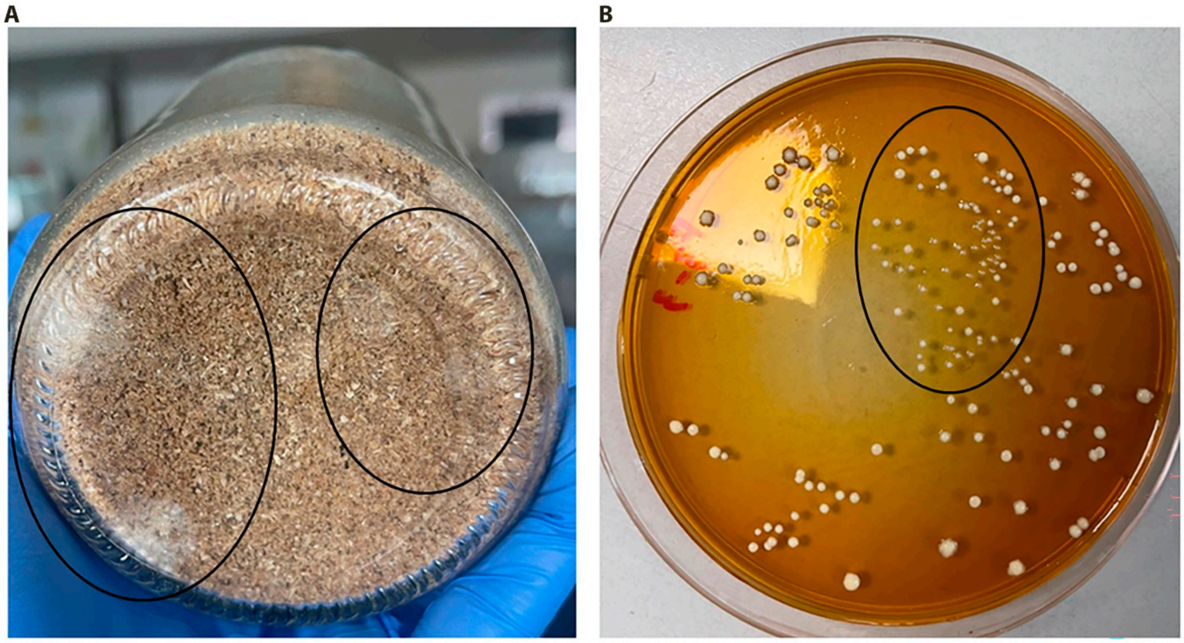

Figure S3. Evidence of contamination during the fermentation of brewer's spent grain with *Limosilactobacillus fermentum* on Day 4. (A) Visible fungal growth in the fermentation jar. (B) Morphologically distinct colonies observed on De Man, Rogosa and Sharpe (MRS) agar following plating of the fermentation sample. Both images were captured on Day 4 of fermentation.

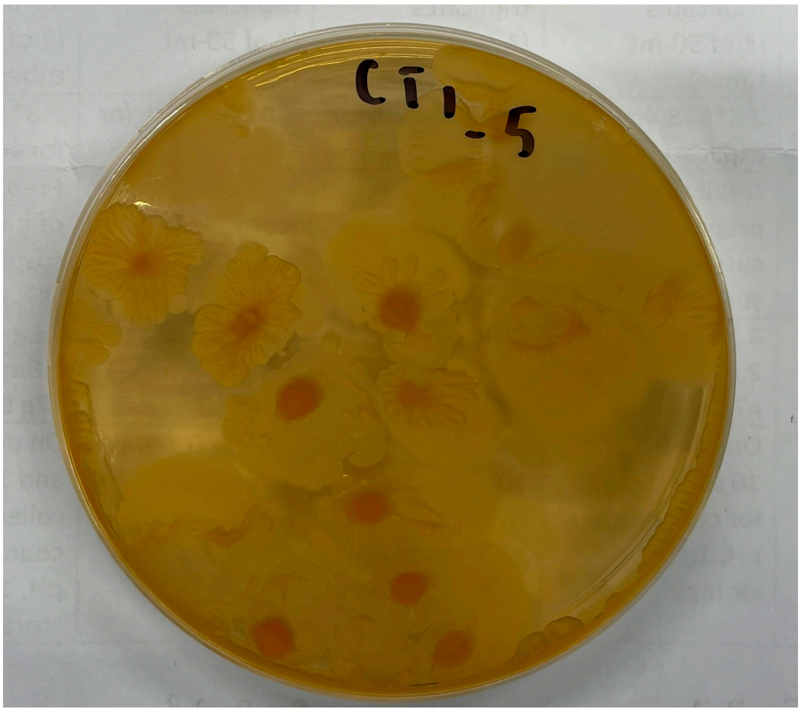

Figure S4. Heavy contamination observed on Day 4 in uninoculated, two-step heat-treated brewer's spent grain (control samples) during the experiments described in Section 3.6.

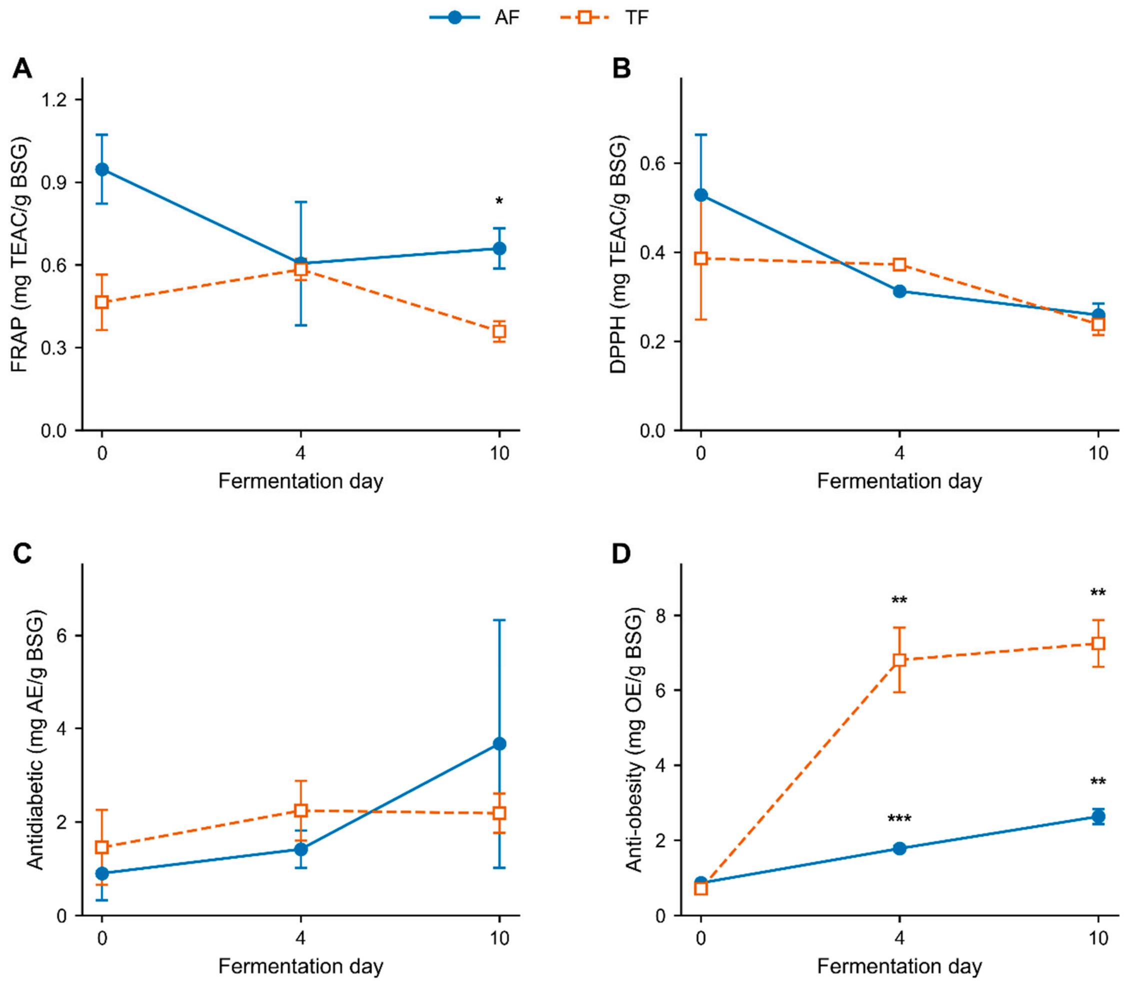

Figure S5. Bioactivity of heat-pretreated brewer's spent grain (BSG) inoculated with *Limosilactobacillus fermentum* during a 10-day fermentation period. (A-B) Antioxidant activity measured by the FRAP and DPPH assays, respectively, and expressed as mg TEAC/g BSG. (C) Antidiabetic activity, expressed as mg AE/g BSG. (D) Anti-obesity activity, expressed as mg OE/g BSG. AF and TF denote autoclaved and two-step heat-treated BSG, respectively. Data are presented as mean ± SD from three independent fermentation replicates (n = 3). Asterisks indicate significant differences relative to Day 0 within the corresponding treatment and assay, determined using Welch's two-sample *t*-test (*$p < 0.05$, **$p < 0.01$, and ***$p < 0.001$).
